# MRI deep learning models for assisted diagnosis of knee pathologies: a systematic review

**DOI:** 10.1007/s00330-024-11105-8

**Published:** 2024-10-18

**Authors:** Keiley Mead, Tom Cross, Greg Roger, Rohan Sabharwal, Sahaj Singh, Nicola Giannotti

**Affiliations:** 1 The University of Sydney School of Health Sciences, Sydney, NSW Australia; 2 The Stadium Sports Medicine Clinic, Sydney, NSW Australia; 3 Vestech Medical Pty Limited, Sydney, NSW Australia; 4 The University of Sydney School of Biomedical Engineering, Sydney, NSW Australia; 5 PRP Diagnostic Imaging, Sydney, NSW Australia

**Keywords:** Knee, Artificial intelligence, Magnetic resonance imaging, Deep learning, Three-dimensional

## Abstract

**Objectives:**

Despite showing encouraging outcomes, the precision of deep learning (DL) models using different convolutional neural networks (CNNs) for diagnosis remains under investigation. This systematic review aims to summarise the status of DL MRI models developed for assisting the diagnosis of a variety of knee abnormalities.

**Materials and methods:**

Five databases were systematically searched, employing predefined terms such as ‘Knee AND 3D AND MRI AND DL’. Selected inclusion criteria were used to screen publications by title, abstract, and full text. The synthesis of results was performed by two independent reviewers.

**Results:**

Fifty-four articles were included. The studies focused on anterior cruciate ligament injuries (*n* = 19, 36%), osteoarthritis (*n* = 9, 17%), meniscal injuries (*n* = 13, 24%), abnormal knee appearance (*n* = 11, 20%), and other (*n* = 2, 4%). The DL models in this review primarily used the following CNNs: ResNet (*n* = 11, 21%), VGG (*n* = 6, 11%), DenseNet (*n* = 4, 8%), and DarkNet (*n* = 3, 6%). DL models showed high-performance metrics compared to ground truth. DL models for the detection of a specific injury outperformed those by up to 4.5% for general abnormality detection.

**Conclusion:**

Despite the varied study designs used among the reviewed articles, DL models showed promising outcomes in the assisted detection of selected knee pathologies by MRI. This review underscores the importance of validating these models with larger MRI datasets to close the existing gap between current DL model performance and clinical requirements.

**Key Points:**

***Question*** *What is the status of DL model availability for knee pathology detection in MRI and their clinical potential*?

***Findings*** *Pathology-specific DL models reported higher accuracy compared to DL models for the detection of general abnormalities of the knee. DL model performance was mainly influenced by the quantity and diversity of data available for model training*.

***Clinical relevance*** *These findings should encourage future developments to improve patient care, support personalised diagnosis and treatment, optimise costs, and advance artificial intelligence-based medical imaging practices*.

**Graphical Abstract:**

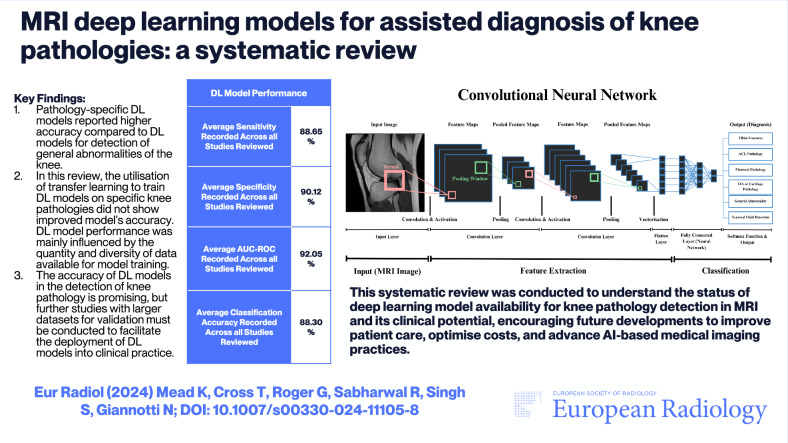

## Introduction

Knee injuries are a prevalent health concern globally, affecting both paediatric and adult populations [1]. Current diagnosis involves clinical examination followed by imaging such as X-ray and magnetic resonance imaging (MRI) [2]. MRI is crucial in diagnosing knee injuries due to its superior soft tissue contrast resolution [3]. In-plane two-dimensional (2D) MRI knee studies are typically acquired in three orthogonal planes using a combination of sequences. While 2D knee MRI is useful for assessing various conditions, its limitations include restricted spatial resolution, partial volume effect (PVE), and geometrical constraints that may limit a thorough interrogation of selected small features of knee injuries [4]. Additionally, 2D MRI does not allow image reconstruction onto arbitrary anatomical planes [5].

Advanced MRI sequences like three-dimensional (3D) MRI can gather larger data sets offering additional information compared to 2D MRI [6]. 3D MRI sequences capture data volumes with higher spatial resolution and reduced PVE [7]. Moreover, it enables image reconstruction on arbitrary diagnostic planes. Despite the radiologist’s preference for multi-sequence 2D MRI due to their enhanced contrast-to-noise ratio (CNR), the traditional limitations of 3D MRI including longer scan times are now mitigated through high magnetic field scanners [8] and compressed sensing techniques that expedite scan time [9]. Furthermore, the recent application of deep-learning (DL) denoising algorithms into 3D MRI sequences has promised to deliver excellent improvements in CNR.

Interpreting knee MRI images requires significant experience [10]. While proficient radiologists exhibit good diagnostic accuracy in knee MRI exams, attaining such expertise demands rigorous training [11]. Artificial intelligence (AI) has recently emerged as a transformative force in the field of medical imaging [12]. DL, a sub-field of AI, is capable of leveraging advanced image pattern recognition capabilities to detect abnormalities and has the potential to revolutionise the way we approach MRI analysis and the classification of injuries and diseases [13]. Today, a growing number of DL models for radiology applications are being developed using different convolutional neural network (CNN) infrastructures, training and validation techniques.

In the context of knee MRI, recent studies have demonstrated the feasibility of training DL models with MRI data to help clinicians with limited expertise in assessing knee injuries [14]. Nevertheless, the precision of DL models for diagnosis remains under investigation. This systematic review aims to summarise the status of the DL MRI models developed for the classification and assisted diagnosis of common knee injuries and diseases.

## Materials and methods

### Protocol

Ethics approval was deemed unnecessary by the Research Integrity and Ethics Committee at the University of Sydney. A retrospective systematic review was conducted following protocols outlined in The Joanna Briggs Institutes’ Manual for Evidence Synthesis [15]. The manuscript structure adhered to the preferred reporting items for systematic reviews and meta-analyses (PRISMA) checklist [16]. Articles were independently screened by title, abstract and full text by the two independent reviewers.

### Search strategy

We searched five online databases (SCOPUS, Pubmed, Web of Science, Science Direct, and Cochrane) from January 1, 2013, to May 12th 2024, using English terms: (magnetic resonance OR magnetic resonance imaging OR MRI OR MR) AND (knee) AND (deep learning OR DL) AND (3D) (see Appendix A).

### Inclusion and exclusion criteria

Articles published in the last eleven years between 2013 and 2024, in English, and peer-reviewed involving adult human participants were included if they discussed DL models assessing pathology in knee MRI images. Exclusions comprised studies unrelated to knee MRI, focusing on modalities other than MRI, or solely on DL segmentation without injury classification and diagnosis. Review papers were excluded. Articles that did not focus on the detection of pathologies, such as only segmenting knee structures, were excluded. Object detection in MRI studies such as the localisation of structures by AI models, volumetric anatomy calculations, grading and severity staging, pathology differentiation, and progression predictions were not included in pathologies as this study is focussed solely on the detection of knee pathologies and injuries. Studies that focussed on non-human or paediatric populations were also excluded.

### Data screening

The covidence platform facilitated duplicate removal, while both reviewers independently screened titles and abstracts of the 1884 publications. Full-text review discussions resolved discrepancies between the two reviewers, ensuring consensus on study selection and data extraction.

### Extraction

The data extraction method utilised a closed-question format established pre-study with a custom-built data extraction template (see Appendix B). Two reviewers independently charted data in Covidence using the template. Disagreements were resolved through discussions, updating the form iteratively to accommodate study variations.

### Synthesis of results

The results obtained from Covidence were downloaded and standardised to ensure consistency in reporting metrics. For studies that provided ranges or multiple sets of data, such as separate performance metrics for 2D and 3D MRI DL models or different versions of the same model, the highest-performing model was selected for analysis. Consequently, to evaluate the performance of DL models, this study conducted several subgroup analyses, including comparisons based on the type of MRI scans used, pathology-specific performance, type of CNNs used, internal versus external datasets, the impact of transfer learning, and the types of ground truths used, categorised by pathology. For each subgroup, performance metrics such as sensitivity, specificity, area under the receiver operating characteristic curve (AUC-ROC), and accuracy were extracted when available and then averaged across the studies to identify trends. Specifically, this study calculated the mean values for sensitivity, specificity, AUC-ROC, and accuracy for each subgroup.

## Results

**Fig. 1:**
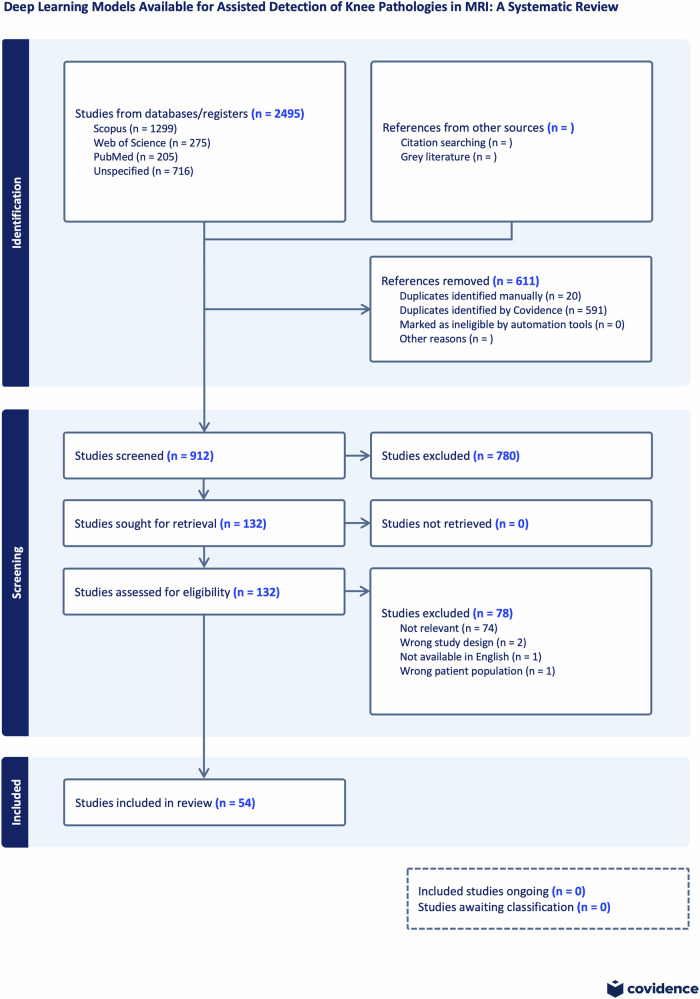
PRISMA flow diagram showcasing the selection of evidence for the systematic review ‘DL models available for assisted detection of knee pathologies in MRI’

The initial online search performed on the 12th of May 2024 yielded 1884 articles excluding duplicates. A total of 54 articles fulfilled the inclusion criteria and progressed to full-text analysis (Fig. 1). A descriptive summary of reported results was presented in data charting tables (see Appendix C).

### General study information, pathology, and article data sources

Among 54 studies analysed, 94% (*n* = 51) [14, 17–66] were retrospective cohort studies while comparative analyses made up 4% (*n* = 2) [67, 68] and diagnostic test accuracy studies made up 2% (*n* = 1) [69] of the selected studies. Anterior cruciate ligament (ACL) injuries were the focus of 35% (*n* = 19) of articles [14, 19, 24, 32, 34, 35, 37, 42, 45, 47, 48, 52, 53, 57, 58, 62, 64, 65], followed by meniscal pathologies in 24% (*n* = 13) [20, 22, 27, 28, 33, 40, 41, 46, 55, 56, 59, 61, 70], general abnormalities in 20% (*n* = 11) [23, 26, 30, 31, 39, 43, 54, 60, 66–68], and osteoarthritis (OA) or cartilage pathologies in 17% (*n* = 9) [17, 18, 21, 25, 36, 38, 48, 50, 51]. Synovial fluid detection (*n* = 1, 2%) [29], and tibial fractures (*n* = 1, 2%) [44] were less frequent. More than half of the studies (*n* = 29, 54%) used local or private databases [19, 22–24, 27, 29, 32–36,40–42, 44, 48–52, 55, 58, 59, 61–66], while the rest employed open-access databases like MRNet (*n* = 9, 17%) [31, 43, 45, 46, 53, 54, 57, 67, 68] and the dataset (*n* = 6, 11%) [17, 18, 20, 21, 25, 56], which reported almost identical performance regardless of where the data was sourced from in performance metrics including sensitivity, specificity, AUC-ROC and accuracy.

### MRI data

In the systematic review of 54 studies, MRI scanner specifications varied widely. Twenty-one studies (39%) did not specify the scanner used [14, 17, 24–27, 29–31,37, 38, 43–46, 48, 49, 53, 54, 68, 69], while 19 (35%) employed 3-Tesla (3-T) machines [18–23, 28, 34, 36, 39–41, 47, 50, 51, 56, 60–62] (Table 1). Seven studies (13%) utilised both 1.5-Tesla (1.5-T) and 3-T machines [35, 42, 52, 57–59, 67], five (9%) used 1.5-T machines [32, 33, 63, 64, 66], and two (4%) employed 1-Tesla (1-T), 1.5-T, and 3-T machines [55, 65] (Table 2). Thirty-one studies (57%) utilised 2D MRI [19, 22–24, 27, 29, 30, 32–37, 40–42, 45, 47, 48, 50, 52, 55, 57–59, 61, 63–67], while eight (15%) employed 3D MRI [17, 18, 20, 21, 28, 39, 60, 62]. Three studies (6%) used both 2D and 3D MRI [25, 51, 56], and twelve studies (22%) did not specify the type of MRI data used [14, 26, 31, 38, 43, 44, 46, 49, 53, 54, 68, 69]. MRI sequences varied greatly, with sagittal images alone used in twenty-one studies (39%) [17–21, 23, 27, 34, 36–38,41, 45, 48, 50, 52, 53, 61, 63, 64, 69], 3D volumes in seven studies (13%) [14, 28, 39, 51, 58, 60, 62], and combinations of sagittal, coronal, and axial images in eleven studies (20%) [24, 26, 30, 31, 43, 46, 49, 54, 57, 66, 67]. Eight studies (15%) used sagittal and coronal images together [25, 29, 33, 40, 42, 55, 59, 65].

**Table 1:** An overview of the number of studies including in the systematic review that utilised various MRI machine characteristics including the magnet strength, data type, and plane utilised for image analysis with DL models

MRI machine characteristics	Sub-set of data	Number of studies included in the review with this sub-set of data, (*n* = )	Percentage of overall studies included in the review with this sub-set of data, (%)
MRI machine magnet strength	1.5 T only	4	8%
	3 T only	19	36%
	Combination of 1.5 T and 3 T	7	13%
	Combination of 1 T, 1.5 T and 3 T	2	4%
	Not Listed	21	40%
MRI data type	2D only	30	57%
	3D only	8	15%
	Combination of 2D and 3D	3	6%
	Not listed	12	23%
MRI plane utilised in image analysis	Sagittal only	21	40%
	Coronal only	2	4%
	Axial only	0	0%
	Oblique sagittal only	1	2%
	3D sequence only	7	13%
	Combination of coronal and sagittal	8	15%
	Combination of sagittal, axial and coronal	10	19%
	Combination of sagittal, and 3D	1	2%
	Combination of sagittal, coronal, axial and 3D	1	2%
	Not listed	2	4%

**Table 2:** An overview of the number of studies including in the systematic review that utilised various data augmentation methods to expand their dataset of MR images of the knee for DL model analysis

Data augmentation use	Sub-set of data	Number of studies included in the review with this sub-set of data, (*n* = )	Percentage of overall studies included in the review with this sub-set of data, (%)
Studies reviewed that used data augmentation	Single technique*	2	4%
	Combination of techniques*	22	42%
	Unspecified technique(s)	1	2%
Studies reviewed that did not use data augmentation	N/A	5	9%
Studies reviewed that did not specify if data augmentation was used	N/A	23	43%

* For a complete list of data augmentation techniques, please refer to Appendix C

### CNN architecture and data processing

ResNet was the primary CNN architecture used in 11 studies (19%) [18, 19, 22, 31, 35, 41, 56, 61, 64, 68, 69], followed by VGG in 6 studies (11%) [32, 36, 38, 43, 54, 67], DenseNet in four studies (8%) [17, 34, 49, 58], DarkNet in three studies (6%) [40, 53, 63]. Sixteen studies (30%) did not enlist an existing CNN architecture and created a customised DL model [21, 23, 24, 27, 28, 31, 37, 39, 42, 44, 45, 47, 55, 59, 67]. Data augmentation was utilised in 26 studies (48%) [17, 19, 20, 22, 23, 25, 27, 29–32, 39, 41, 43, 47, 50, 52, 53, 56, 58, 60, 62, 64, 66–68], 23 studies did not report whether data augmentation was used (43%) [14, 18, 21, 28, 33–38, 40, 42, 44, 48, 49, 51, 54, 57, 59, 61, 63, 65, 69] and five studies did not use data augmentation (9%) [24, 26, 45, 46, 55] (Table 1). The details of the several data augmentation methods are available in Appendix C along with the details of the datasets used. Thirty-nine studies (72%) conducted a validation process of their DL models against previously unseen data [17, 18, 21–24, 27–31, 34–42, 46, 47, 50, 52, 53, 55–62,64–67, 69, 70]. Of these, thirty-two used internal validation data (82%) [17, 18, 21–23, 28–31, 34–39, 42, 46, 47, 50, 52, 53, 56–59, 61, 62, 64–66, 69, 70], four used both internal and external validation data (10%) [40, 41, 55, 67], and three used external data only (8%) [24, 27, 60] (Table 3). Transfer learning was utilised in eighteen of the studies (33%) [17–19, 22, 26, 29, 30, 35, 38, 46, 48, 53, 54, 57, 62, 66, 68, 69] included from pre-existing datasets such as ImageNet, and we found that the performance metrics of DL models that used transfer learning reported specificity, AUC-ROC, accuracy and sensitivity of 0.896, 0.916, 0.871, and 0.925, respectively compared to no transfer learning use 0.903, 0.922, 0.889, and 0.875, respectively (see Appendix D).

**Table 3:** The average values for four selected performance metrics (specificity, AUC-ROC, accuracy, and sensitivity) were recorded for each type of validation across all studies

Type of validation	Average specificity value	Average AUC-ROC value	Average accuracy value	Average sensitivity value
Internal (*n* = 31)	0.899	0.924	0.880	0.880
External (*n* = 3)	0.890	0.926	Not measured	0.850
Both internal and external (*n* = 4)	0.897	0.914	0.885	0.842
Not listed (*n* = 15)	0.933	0.905	0.886	0.939

Note that if the performance metric was not listed, it was not included in the average

### Performance outcomes and ground truth references

**Fig. 2:**
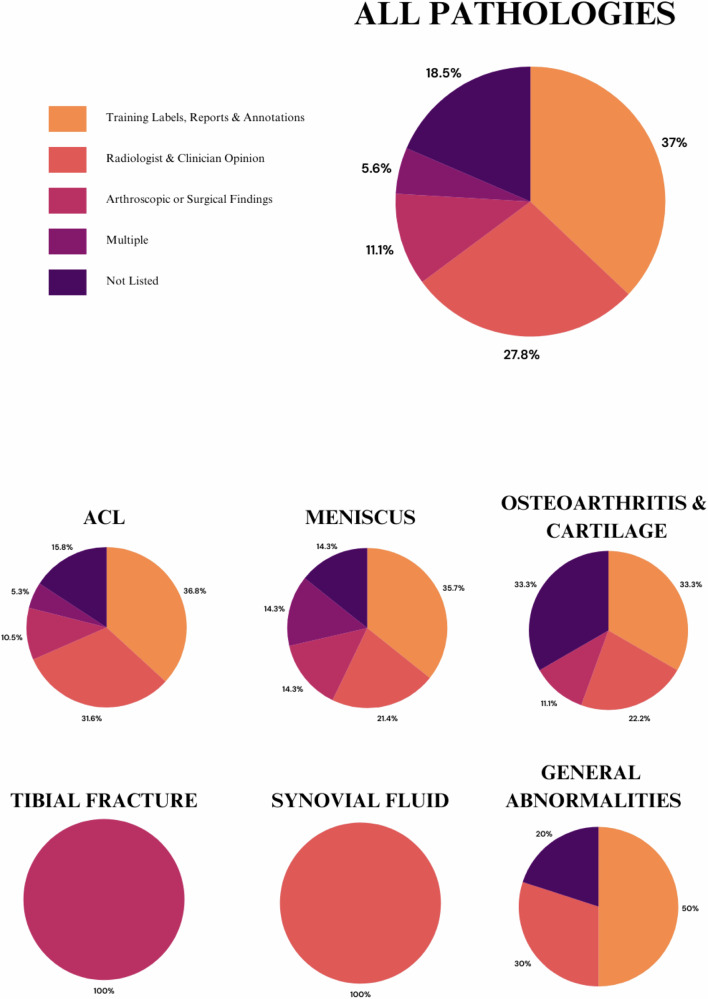
Graphical depiction of the occurrence of various ground truth or reference standards representative of the articles analysed in the review

Ground truth for knee pathologies in the studies analysed included training labels, reports, or annotations (*n* = 20, 37%) [18–21, 27, 30, 31, 37, 38, 41, 43, 46, 49, 57,63–65, 67–69], radiologist or clinician opinion (*n* = 15, 28%) [14, 23, 28, 29, 32, 35, 36, 39, 50, 52, 53, 55, 60, 62, 66], arthroscopic or surgical findings (*n* = 6, 11%) [34, 40, 44, 51, 58, 59], or a combination of physician opinion with arthroscopic or surgical findings, or training labels, reports or annotations (*n* = 3, 6%) [22, 42, 59]. Ten studies did not list their ground truth (19%) [17, 24–26, 33, 45, 47, 48, 54, 56] (Fig. 2). The articles that did not list a ground truth reported higher performance metrics compared to articles that utilised a ground truth or reference standard including sensitivity, specificity, AUC-ROC and accuracy (see Appendix E).

DL model performance outcomes were averaged across knee pathologies for sensitivity, specificity, AUC-ROC, and classification accuracy, reporting 88.65%, 90.12%, 92.05%, and 88.30%, respectively. Specific knee abnormality training improved outcomes, with average specificity, AUC-ROC, accuracy, and sensitivity of 90.57%, 92.72%, 88.91%, and 88.67%, respectively. General abnormalities MRI studies showed averages of 86.51%, 89.80%, 84.48%, and 88.48%, respectively, highlighting specialised model benefits of up to 4.5% (Table 4). 2D MRI data yielded higher model performance averages: specificity, accuracy, and sensitivity of 0.904, 0.898, and 0.892, compared to 3D MRI data at 0.881, 0.852 and 0.870. However, the AUC-ROC performances for combined 2D and 3D MRI data-based models averaged 0.934, compared to 0.931 and 0.871 for 2D data alone and 3D data alone, respectively. No DL models gained regulatory approval.

**Table 4:** The average values for four selected performance metrics (specificity, AUC-ROC, accuracy and sensitivity) recorded for each knee injury of pathology across all studies

Performance Factor	Sub-Set of Data	Average Specificity Value	Average AUC-ROC Value	Average Accuracy Value	Average Sensitivity Value
Pathology Focus	ACL Injuries (*n* = 19)	0.937	0.960	0.911	0.932
	Meniscal Injuries (*n* = 13)	0.869	0.893	0.863	0.809
	General Abnormalities (*n* = 10)	0.865	0.893	0.845	0.868
	Osteoarthritis or Cartilage Pathologies (*n* = 9)	0.887	0.924	0.883	0.874
	Synovial Fluid Detection (*n* = 1)	0.821	Not measured	0.868	0.893
	Tibial Fractures (*n* = 1)	0.932	Not measured	0.953	0.969

Note that if the performance metric was not listed, it was not included in the average

## Discussion

### Datasets

This systematic review highlights the reliance on large datasets for developing DL models, primarily from retrospective cohort studies. Today, imaging facilities and healthcare services are under a significant workload and may lack the logistical capacity, time, and resources to undertake prospective MRI studies aimed explicitly at DL model development. Consequently, 94% of the studies analysed utilised existing data. Of the currently available open-source datasets, the MRNet database featured prominently, appearing in approximately 17% of the studies, offering diverse DL model performance outcomes. Extending open-source MRI datasets holds profound significance in advancing the development of DL models for assisted diagnosis in healthcare. These datasets serve as invaluable resources for researchers, clinicians, and developers worldwide, enabling them to access diverse and comprehensive collections of medical images crucial for training and validating DL algorithms. Furthermore, increased accessibility to diverse MRI datasets encourages collaboration and innovation across the medical imaging community, facilitating the exploration of novel methodologies and techniques for improved diagnostic accuracy and patient care.

Despite some DL models exhibiting versatility in detecting various knee pathologies and general abnormalities, the DL models tailored for a specific task—ACL injury detection for instance—demonstrate superior performance compared to those designed for broader abnormality detection. Recent advances in DL technology have now enabled the use of transfer learning in the context of medical imaging. Transfer learning allows the knowledge acquired from one specialised task, such as ACL injury detection, to be leveraged and transferred to additional related tasks. In this case, DL models initially trained to excel in identifying a particular knee pathology could serve as valuable foundations for the development of a more comprehensive knee injury detection multi-model structure capable of assessing with high accuracy a large range of diseases.

### MRI

The variability in the datasets due to the use of different MRI techniques presents a significant challenge for the clinical deployment of DL models. This issue is particularly pronounced due to the varying image quality that results from differences in MRI scanner field strengths. Studies have shown that 3-T MRI scanners provide superior image quality, especially for OA and ACL imaging [70], nonetheless, the utilisation of diverse MRI protocols further complicates the matter. A standard MRI examination of the knee typically involves the acquisition of a range of sequences in three orthogonal imaging planes. Despite this, the specific protocol may vary significantly based on patient indications and site-specific factors. For example, indications of ACL injury on MRI requests may prompt the inclusion of thin and ultra-thin oblique sagittal or coronal MRI, which is demonstrated to enhance the diagnostic accuracy for ACL injuries [71, 72]. This diversity in imaging techniques hampers creating universally effective DL models for real-world clinical use. Standardising imaging techniques or training models with more diverse real-world data could improve DL model deployment. In the studies analysed, the MRI sequences acquired in the sagittal plane were the most used to train DL models. This is most likely due to its ability to see the ACL in its entirety compared to other imaging planes [73], and it should be noted that the ACL injury was the most relevant pathology in the studies included in our review. We believe that the preference for 2D MRI over 3D MRI data in DL model development may be due to their acceptance in standard MRI protocols and availability in repositories like MRNet, however, for indications such as ACL injuries, the ability to interrogate the “rupture zone” in high resolution on patient’s acute and follow-up MRI examinations for ACL-related pathologies is essential for treatment planning with emerging treatments such as the cross bracing protocol [74]. Future research should explore the potential impact of 3D MRI data on diagnostic accuracy and DL model performance as 3D MRI gains acceptance in medical imaging clinics.

### DL model development

DL models require large amounts of data to achieve a high level of accuracy for disease classification and therapeutic management prediction purposes [75]. In the absence of large dataset availability, data augmentation is a method often employed in AI studies to increase the available data [61]. In the studies analysed, 48% of studies stated that data augmentation was used. Data augmentation methods increased models’ performance by increasing the number of images for DL model training. One study reported an AUC-ROC of 0.905 [31], whereas a 2021 study utilising similar CNN (ResNet) and database (MRNet) reported an AUC-ROC of 0.8196 [46], with the primary difference between the two studies being the utilisation of data augmentation. The use of data augmentation to increase the available data was able to aid in improving performance outcomes, suggesting that the performance of models is often dependent on the quantity of data available for training, testing and validation purposes.

The main CNN used was ResNet in 19% of studies, however, a wide variety of different architectures were used for the models in the articles analysed. The type of CNN used can affect the performance of the DL model. In 2022 study that utilised the MRNet dataset opted to use the CNN, ‘Inception-v3’ and reported performance outcomes of 0.9634, 0.9542, and 0.9513 for specificity, accuracy and sensitivity, respectively [57]. A similar study conducted in 2021 that used the same MRNet dataset but utilised the ResNet50 CNN reported significantly lower performance outcomes for specificity, accuracy, and sensitivity [46]. Whilst transfer learning was utilised in some studies, it did not significantly improve the performance of these models, with models that did not use transfer learning outperforming those that did in specificity, AUC-ROC, and accuracy metrics. Future research is required to determine if models that employed transfer learning were impacted by the training of the model, or if the performance of models is more dependent on the construction of the DL model.

The 2D MRI models showed higher average specificity, AUC-ROC, accuracy, and sensitivity compared to the 3D MRI models. This suggests that model performance may be influenced by the algorithm used and the abundance of input data, as 2D MRI data are generally more prevalent and thus may lead to better-trained models. When comparing DL models developed with 2D and 3D MRI data, the results showed a higher individual data type AUC-ROC compared to the combined data type accuracy that appeared to improve. This discrepancy between AUC-ROC and accuracy metrics may due caused by the differences in how these metrics are calculated. Furthermore, we observed that the variability in ground truth establishment across studies likely influences these differences. The combined imaging data approach with expert ground-truth annotation will provide a more robust foundation for the validation of DL models suitable for both data types.

The ground truth, used as a reference standard, assessed knee pathologies in the studies analysed to benchmark DL model performance against human performance. The potential to conduct clinical testing of DL models is supported by the recent encouraging findings that showed high DL model accuracy compared to interpretations made by expert clinicians. Outcome measures varied across studies, depending on DL model aims and inclusion/exclusion of segmentation aspects. Performance metrics like sensitivity, specificity, AUC-ROC, precision, and classification accuracy were averaged across knee pathologies. Two-dimensional MRI data showed higher average specificity, AUC-ROC, accuracy, and sensitivity than those using 3D MRI suggesting that model performance may be influenced by the algorithm and input data abundance. The combined 2D and 3D data reported a higher AUC-ROC compared to individual MRI data types, potentially due to the AUC-ROC calculation’s consideration of sensitivity and specificity unlike accuracy calculations, and its comparable performance to 2D MRI alone.

Notably, the lack of approval of any models included in the studies utilised in the review by regulatory bodies such as the Food and Drug Administration or international counterparts, suggests there remain limitations to these models that inhibit their clinical implementation. Despite some studies showing high classification accuracy, the DL models have not been clinically applied, raising concern about their performance in a real-life setting. AI in the healthcare setting comes with ethical, financial, and legal implications that require a high level of consideration at academic, clinical, industrial and government levels. Whilst the potential of AI for clinical use continues to be debated by the medical community, especially regarding concerns about the displacement of radiologists, the lack of regulatory body approval hinders these models from being deployed clinically. Prospective studies comparing multiple DL model performance to radiologist performance will be able to determine if DL models are best utilised as an alternative to radiologists or as an assistive tool. Ideally, current DL models can be trained with a more robust dataset from varying vendors, magnet strengths and sequences prior to future research determining if they are suitable for clinical deployment. Furthermore, DL models have the potential to aid not only radiologists but also other healthcare professionals regardless of their radiology experience.

### Limitations

The rapid development of AI in medical imaging means newer articles may now exist. The primary challenge found while reviewing articles was the inconsistency in how the data was reported. In 2020, the Checklist for Artificial Intelligence in Medical Imaging (CLAIM) was developed, promoting “clear, transparent, and reproducible scientific communication about the application of AI to medical imaging” and becoming the clinical standard for “best practice” [60], however, the checklist has now been amended to ensure wider adoption to solve the problems initially reported [76]. Alternatively, to CLAIM, checklists including STARD-AI (standards for reporting of diagnostic accuracy study-AI), CONSORT-AI (consolidated standards of reporting trials-AI), SPIRIT-AI (standard protocol items: recommendations for interventional trials-AI), FUTURE-AI (fairness universality traceability usability robustness explainability-AI), MI-CLAIM (minimum information about clinical artificial intelligence modelling), MINIMAR (minimum information for medical AI reporting), and RQS (radiomics quality score) [77], remains available for use to provide structure to projects. In lieu of the CLAIM checklist, or other recommended checklists being applied consistently across the 54 studies included in the review, the wide variation of methodologies and aims of studies makes a robust comparison of DL models challenging to conduct. Due to the insufficiency in data reporting, the highest-performing model tested was included to demonstrate the potential and reality of AI performance to date with optimal parameters despite testing multiple backbones and models on various factors such as multiple or single slices, and on one or more pathologies. Additionally, many studies did not report on the loss functions or error estimates when reporting on the accuracy of DL models which we acknowledge as a limitation of this study. Additionally, out of the included studies, 14 (26%) did not provide details on their ground truths. Recognising the potential for bias and the scepticism warranted in such cases, we conducted an additional sub-analysis to examine the impact of excluding these studies. Our findings indicate that the studies without listed ground truths reported higher performance metrics, including sensitivity, specificity, AUC-ROC, and accuracy, compared to those that utilised a defined ground truth or reference standard. This discrepancy suggests that the absence of a clearly defined ground truth might lead to inflated performance claims, either due to less rigorous validation methods or potential biases in reporting. These findings underscore the necessity for transparency and rigour in reporting ground truths in DL studies. Without a reliable reference standard, the validity of the reported accuracy may be questionable, and conclusions drawn from such studies should be approached with caution. The higher performance metrics in studies lacking ground truths may reflect an overestimation of model capabilities, leading to potential misguidance in clinical application. Despite these challenges, articles with varying performance information were included to provide a comprehensive review of available models for assisting knee diagnosis in MRI.

Based on up-to-date knowledge, only two other systematic reviews on DL models in MRI in knee pathologies have been published [78, 79]. However, this study provides an overview of the models available and their accuracy on a broad scale. This review’s novelty lies in its inclusion of additional conditions, sub-group analyses revealing new information, and consideration of 3D MRI and how this can be applied to DL models.

### Future directions

Whilst many studies that demonstrated high diagnostic accuracy do not comment on potential reasons for their lack of clinical deployment, an example of a common factor was the need for additional validation or larger testing datasets, such as in “future directions include further algorithm development on expanded datasets for comprehensive evaluation of sports-related musculoskeletal pathologies” [35]. Currently, no DL models are clinically deployed to aid in detecting knee abnormalities on MRI. Future research should prioritise generating abundant 2D and 3D MRI data tailored to specific pathologies for transfer learning to develop versatile DL models. Open-access data sharing will enhance data availability, improving DL model performance through better training and validation processes.

## Conclusion

This systematic review highlights that the fine-tuning of DL models specific to knee pathologies can be used to improve model performance compared to general screening models. This progress should be further solidified through the execution of more extensive validation studies aimed at enhancing the overall DL models’ performance, and a prospective study investigating if the DL models investigated are suitable to be utilised as an assistive tool in the clinical setting.

## Supplementary information

ELECTRONIC SUPPLEMENTARY MATERIAL

## Abbreviations

ACL: Anterior cruciate ligament
AUC: Area under curve
AI: Artificial intelligence
CLAIM: Checklist for artificial intelligence in medical imaging
CNR: Contrast-to-noise ratio
CNN: Convolutional neural network
DL: Deep learning
MR: Magnetic resonance
MRI: Magnetic resonance imaging
OA: Osteoarthritis
PVE: Partial volume effect
PRISMA: Preferred reporting items for systematic review and meta-analysis
ROC: Receiver operating curve
3D: Three-dimensional
2D: Two-dimensional
