# Supplementary material for: MRI deep learning models for assisted diagnosis of knee pathologies: a systematic review
Source: Eur Radiol. 2024 Oct 18;35(5):2457–69. doi: 10.1007/s00330-024-11105-8 (PMC12021734; doi:10.1007/s00330-024-11105-8)
Supplement: Supplementary file 1 — ELECTRONIC SUPPLEMENTARY MATERIAL [file 330_2024_11105_MOESM1_ESM.pdf]

MRI Deep Learning Models for Assisted Diagnosis of Knee Pathologies: A Systematic Review

ELECTRONIC SUPPLEMENTARY MATERIAL

Appendix A

The Search Strategy Utilised in this Systematic Review

Online Database Search in SCOPUS, Pubmed, Web of Science, Science Direct and Cochrane (1st January 2013 until 7th April 2023)

**#1**	"Magnetic Resonance Imaging" [Mesh] OR "Magnetic Resonance" OR "MRI" OR "MR"
**#2**	"Knee" [Mesh]
**#3**	"Deep Learning"[Mesh] OR "DL"
**#4**	Three-dimensional [Mesh] OR "3D"
**#5**	**#1** AND **#2** AND **#3** AND **#4**

Appendix B

Data Extraction Template Used in Covidence

PREVIEW

General info

Title

Title of paper / abstract / report that data are extracted from

First author

Year

Country in which the study conducted

- United States
- UK
- Canada
- Australia
- Other

Study design

- Randomised clinical trial
- Retrospective cohort study
- Prospective cohort study
- Systematic review
- Diagnostic test accuracy study
- Clinical prediction rule
- Economic evaluation
- Other

Disease (topic of the study)

Aim of study

Follow up

- Yes
- Other

FDA/TGA/EC mark approved

Methods "general section"

Data source (local or open-source register)

- Local
- Other

Population description

Inclusion criteria

Exclusion criteria

Methods "imaging section"

MRI machine used to acquire data (magnetic field + vendor)

MRI sequences used

MRI images used (i.e. axial, sagittal, 3D volumes)

Image reconstruction

Methods "deep learning"

Machine learning type

Coding interface used in data processing

(Convolutional) Neural Network used

Number of data for training

Data augmentation used

- Yes
- Other

If yes, what type of data augmentation technique was used?

Was a validation performed

- Yes
- Other

If yes, number of data for validation

Internal or external validation

Definition of ground truth reference standard (i.e. supervised learning by radiologist)

Statistical tests run

Outcomes measures (metrics of model performance i.e. ROC, performance, sensitivity, specificity, Dice coefficient)

Table preview

	Value	p value
Specificity		
AUC-ROC		
Accuracy		
Sensitivity		
Dice coefficient		
F1 Score		
Precision		

Appendix C

Complete Excel Table of Data Extracted from the Studies Included in the Systematic Review

**Covidence #**	2391	2360	2330
**Study ID**	Wang 2023	Yeoh 2023	Voinea 2023
**Title**	Detection algorithm of knee osteoarthritis based on magnetic resonance images	Transfer learning-assisted 3D deep learning models for knee osteoarthritis detection: Data from the osteoarthritis initiative	Detection and Classification of Knee Ligament Pathology based on Convolutional Neural Networks
**Reviewer Name**	Consensus	Consensus	Consensus
**Title**	Detection Algorithm of Knee Osteoarthritis Based on Magnetic Resonance Images	Transfer learning-assisted 3D deep learning models for knee osteoarthritis detection: Data from the osteoarthritis initiative	Detection and Classification of Knee Ligament Pathology based on Convolutional Neural Networks
**First author**	Xin Wang	Pauline Shan Qing Yeoh	»òtefan-Vlad Voinea
**Year**	2023	2023	2023
**Country in which the study conducted**	Other: China	Other: Malaysia & China	Other: Romania
**Study design**	Retrospective cohort study	Retrospective cohort study	Retrospective cohort study
**Disease (topic of the study)**	Osteoarthritis or Cartilage Pathologies	Osteoarthritis or Cartilage Pathologies	ACL Pathology
**Aim of study**	Detect and judge the presence of knee OA.	To fill the void by leveraging transfer learning of 2D pre-trained weights in the 3DCNN in medical imaging, particularly for knee OA diagnosis	To study the usage of pre-trained convolutional neural networks with residual connections (ResNet), in conjunction with image processing techniques to detect ACL pathology and distinguish between multiple tear levels.
**Follow up**	Other: Not listed.	Other: Not listed.	Other: No.
**FDA/TGA/EC mark approved**	No.	No.	No.
**Data source (local or open-source register)**	Other: OAI-ZIB dataset	Other: OAI dataset	Local
**Population description**	60 healthy and 60 diseased knee OA subjects.	Not listed - can be found with OAI dataset.	952 exams acquired by the Faculty of Medicine from Craiova - Imaging Center throughout 2020, 2021 and the first 5 months of 2022, through magnetic resonance analysis.<br><br>The data distribution among the 3 classes was highly unbalanced:<br>‚óè 65 completely torn ACLs<br>‚óè 674 partially torn ACLs<br>‚óè 213 normal ACLs<br>In order to overcome this deficiency that normally prevents the model to generalize and rather overfit the presented data, learning the 43 completely torn ACLs instead of the features that define a torn ACL, the unbalanced class of completely torn ACLs was oversampled to level the 3 classes.<br>The distribution among genders was not balanced, approximately 37% of the subjects being females:<br>‚óè 353 female exams<br>‚óè 599 male exams

			The prevalence of ACLs injuries is generally thought to be<br>the opposite, more on female than male subjects. The gender<br>was considered not to be a defining factor in this study, so the<br>dataset was left unchanged in this regard. Data was quite well<br>balanced when it comes to laterality, 512 left knee exams and<br>440 right knee exams.
**Inclusion criteria**	Not listed.	Not listed.	Not listed.
**Exclusion criteria**	Not listed.	Not listed.	Not listed.
**MRI machine used to acquire data (magnetic field + vendor)**	Not listed.	3T Seimens	3T Philips Ingenia
**MRI sequences used**	3D DESS	3D double echo steady state (DESS)	T2
**MRI images used (i.e. axial, sagittal, 3D volumes)**	Sagittal only.	Sagittal	Sagittal
**Image reconstruction**	Not listed.	Not listed.	Not listed.
**Machine learning type**	CNNs	3D CNNs	CNN
**Coding interface used in data processing**	Tensorflow2.0	Pytorch	Not listed.
**(Convolutional) Neural Network used**	DenseNet	Multiple ie. ResNet, DenseNet, VGG, and AlexNet	ResNet
**Number of data for training**	72/120	70% - 280 MR volumes	N/A
**Data augmentation used**	Yes	Other: Not listed.	Yes
**If yes, what type of data augmentation technique was used?**	Horizontal flipping, image rotation, and offset	N/A	Warp with a random magnitude in interval [-0.25, 0.25]<br><br>Shear with random height and width linear magnitudes in interval [-0.25, 0.25]<br><br>Trapezoid with random height and width magnitudes in interval [-0.25, 0.25]<br><br>Brightness with random beta in interval [-0.3, 0.3]<br><br>Contrast with random alpha in interval [0.7, 2.0]<br><br>Gaussian noise with a standard deviation in interval [0.01, 0.1]<br><br>Gaussian blur with kernel size in range [5, 11] and sigma 0.5
**Was a validation performed**	Yes	Yes	Other: Not listed.
**If yes, number of data for validation**	24/120	20% - 80 MR volumes	Not listed.
**Internal or external validation**	Internal	Internal	N/A
**Definition of ground truth reference standard (i.e. supervised learning by radiologist)**	Not listed.	Training labels, reports, or annotations	Training labels, reports, or annotations
**Statistical tests run**	Not listed.	Accuracy, balanced accuracy, precision, F1 score, AUC	Loss function

**Outcomes measures (metrics of model performance i.e. ROC, performance, sensitivity, specificity, Dice coefficient)**	Accuracy (ACC), Precision (PRE), Sensitivity (SEN), Specificity (SPE), F1-Score, and Matthews Correlation Coefficient (MCC)	Accuracy, balanced accuracy, precision, F1 score, AUC	accuracy, AUC-ROC, F1, precision
**Specificity Value**	0.891		
**AUC-ROC Value**		0.945	0.965
**Accuracy Value**	0.921	0.812	0.871
**Sensitivity Value**	0.960		
**Notes/Comments**	Transfer learning done based on the DenseNet201 model with ImageNet. Results reported as written. There is no comment on clinical applications.	Transfer learning completed using ImageNet. Results shown are based on ResNet18 as the highest performing model. There is no comment on clinical applications and why it has not been implemented however suggests embedding AI tools into devices for assistive purposes.	Transfer learning utilised. Results based on pre-trained RESNET 101 3D model. There is no comment on clinical applications or reasons for lack of clinical deployment.

**Covidence #**	2323	2292	2248
**Study ID**	Berrimi 2023	Yeoh 2023	Ying 2023
**Title**	A 3D Deep Learning Approach for Meniscus Tear Severity at the Region-level	3D Efficient Multi-Task Neural Network for Knee Osteoarthritis Diagnosis Using MRI Scans: Data From the Osteoarthritis Initiative	A deep learning knowledge distillation framework using knee MRI and arthroscopy data for meniscus tear detection
**Reviewer Name**	Consensus	Consensus	Consensus
**Title**	A 3D Deep Learning Approach for Meniscus Tear Severity at the Region-level	3D Efficient Multi-Task Neural Network for Knee Osteoarthritis Diagnosis using MRI Scans: Data from The Osteoarthritis Initiative	A deep learning knowledge distillation framework using knee MRI and arthroscopy data for meniscus tear detection
**First author**	Mohamed Berrimi	Pauline Shan Qing Yeoh	Mengjie Ying
**Year**	2023	2017	2024
**Country in which the study conducted**	Other: France	Other: Malaysia	Other: China
**Study design**	Retrospective cohort study	Retrospective cohort study	Retrospective cohort study
**Disease (topic of the study)**	Meniscal Pathology	Osteoarthritis or Cartilage Pathologies	Meniscal Pathology
**Aim of study**	To detect meniscus tears on MRI images	To investigate the feasibility of multi-task 3D CNN approach for knee OA diagnosis.	To construct a deep learning knowledge distillation framework exploring the utilization of MRI alone or combing with distilled Arthroscopy information for meniscus tear detection.
**Follow up**	Other: Not listed.	Other: No.	Other: Not listed.
**FDA/TGA/EC mark approved**	No.	No.	No.
**Data source (local or open-source register)**	Other: OAI dataset	Other: OAI dataset	Local
**Population description**	Not listed - available in OAI dataset but not clear.	Not listed.	202 patients who had knee MRI examinations and their paired arthroscopic images at Shanghai Sixth People‚Äôs Hospital Affiliated with Shanghai Jiao Tong University School of Medicine between January 2021 and December 2022. The included population consisted of 87 (44.8%) men and 107 (55.2%) women with a mean age of 40.5 years and a standard deviation (SD) of 15.3 years. Five of them had surgical images and MRI exams on both knees (194 patients,

			199 cases). The data composition and utilization of our research are shown in Figure 1. There were 169 injury cases with 60 (30.2%) medial meniscal tears and 125 (62.8%) lateral meniscal tears in the paired knee Arthroscopy-MRI dataset. The report of the knee surgery served as the standard of reference of this study.
**Inclusion criteria**	Not listed.	Not explicitly listed.	See study population above.
**Exclusion criteria**	Not listed.	Not listed.	Patients who had a previous knee surgery history or an interval between the MRI examination and subsequent surgery of more than 3 months were excluded (8 patients)
**MRI machine used to acquire data (magnetic field + vendor)**	3T Seimens Trio	3T Seimens Trio	3.0-T MRI Scanner Philips
**MRI sequences used**	Double Echo Steady-State (DESS) 3D MRI	3D double-echo steady-state (DESS)	proton density (PD)-weighted fat-suppression
**MRI images used (i.e. axial, sagittal, 3D volumes)**	sagittal	Sagittal	Coronal
**Image reconstruction**	Not listed.	Not listed.	Not listed.
**Machine learning type**	3D CNN	CNN	CNN
**Coding interface used in data processing**	Tensorflow	PyTorch	Python 3.9.16 and Torch 1.8.1 + cu111
**(Convolutional) Neural Network used**	3D-Flex Inception	Original	ResNet
**Number of data for training**	Not listed.	280 MRI scans (87 for No OA, 193 for OA)	156-160
**Data augmentation used**	Yes	Other: Not listed.	Yes
**If yes, what type of data augmentation technique was used?**	Specifically, we applied rotations to the volumes, introducing diversity by rotating each slice by an angle of 5%. In addition, we used zooming techniques to focus on specific regions of interest.	N/A	andom rotation, random contrast adjustment and random addition of Gaussian noise to the images
**Was a validation performed**	Other: Not listed.	Yes	Yes
**If yes, number of data for validation**	Not listed.	80 MRI scans (32 for No OA, 48 for OA)	39-40
**Internal or external validation**	Not listed.	Internal.	Internal
**Definition of ground truth reference standard (i.e. supervised learning by radiologist)**	Training labels, reports, or annotations	Training labels, reports, or annotations	Arthroscopic or surgical findings AND Training labels, reports, or annotations
**Statistical tests run**	Not listed.	Precision (PRE), recall (REC), Dice Similarity Coefficient (DSC) and Jaccard Similarity Coefficient (JSC) (for segmentation), precision (PRE), recall (REC)	accuracy, sensitivity, specificity, F1-score and area under the receiver operating characteristic curve (AUC). We chose the threshold that maximized the Youden index for analysis. The performance of the above three models was also evaluated with receiver operating characteristic (ROC) analysis.
**Outcomes measures (metrics of model performance i.e. ROC, performance, sensitivity, specificity, Dice coefficient)**	AUC	Accuracy (ACC), F1-Score.	Specificity, sensitivity, AUC, F1, accuracy
**Specificity Value**			0.787
**AUC-ROC Value**	0.813		0.772

**Accuracy Value**		0.825	0.749
**Sensitivity Value**		0.792	0.750
**Notes/Comments**	No transfer learning listed. Performance metric is determined as an average from the medial and lateral meniscus measurements from their highest performaning customised DL model (3D Flex Inception) in experiment 2 based on meniscal tear detection. There is no comment on clinical applications.	No transfer learning listed. Performance metric is based on OAMTL model. There is no comment on clinical applications.	Transfer learning utilised in the form of a teacher model. Results are based on the distilled student model (teacher-student model) as an average of the medial and lateral meniscus readings. Suggest reason for lack of clinical implementation may be to do with the alck of radiologist input to implementation so far of these models.

**Covidence #**	2242	2235	1482
**Study ID**	Kasuya 2023	Li 2023	Berrimi 2024
**Title**	Feasibility of the fat-suppression image-subtraction method using deep learning for abnormality detection on knee MRI	Automated diagnosis of anterior cruciate ligament via a weighted multi-view network	A semi-supervised multiview-MRI network for the detection of Knee Osteoarthritis
**Reviewer Name**	Consensus	Consensus	Consensus
**Title**	Feasibility of the fat-suppression image-subtraction method using deep learning for abnormality detection on knee MRI	Automated diagnosis of anterior cruciate ligament via a weighted multi-view network	A semi-supervised multiview-MRI network for the detection of Knee Osteoarthritis
**First author**	Shusuke Kasuya	Li Feng	Mohamed Berrimi
**Year**	2023	2023	2024
**Country in which the study conducted**	Other: Japan	Other: China	Other: France
**Study design**	Retrospective cohort study	Retrospective cohort study	Retrospective cohort study
**Disease (topic of the study)**	General Abnormalities	ACL Pathology	Osteoarthritis or Cartilage Pathologies
**Aim of study**	To evaluate the feasibility of using a deep learning (DL) model to generate fat-suppression images and detect abnormalities on knee magnetic resonance imaging (MRI) through the fat-suppression image-subtraction method	To build a three-dimensional (3D) deep learning-based computer-aided diagnosis (CAD) system and investigate its applicability for automatic detection of anterior cruciate ligament (ACL) of the knee joint in magnetic resonance imaging (MRI).	To introduce a semi-supervised multi-view framework and a 3D CNN model for detecting knee OA using 3D Magnetic Resonance Imaging (MRI) scans.
**Follow up**	Other: no.	Other: Not listed.	Other: No.
**FDA/TGA/EC mark approved**	No.	No.	No.
**Data source (local or open-source register)**	Local	Local; Other: In-house MRI-ACL dataset and the publicly available MRNet-v1.0 datase	Other: OAI dataset
**Population description**	The final diagnoses were osteoarthritis (n = 18), meniscal tear (n = 38), ligament tear (n = 5), post-resection of benign tumour (n = 2), Osgood-Schlatter disease (n = 1), and muscle injury (n = 1). In addition, 12 knee MR studies in 6 healthy volunteers who had neither symptoms nor history of trauma in the knee (mean age 34.2 ¬± 9.5 years; 4 males/2 females; 6 right/6 left) were included.	In the MRI-ACL dataset, the retrospective study collects 100 cases, and four views per patient are included. There are 50 ACL patients and 50 normal patients, respectively. The MRNet-v1.0 dataset contains 1,250 cases with three views, of which 208 are ACL patients, and the rest are normal or other abnormal patients.	4796 patients
**Inclusion criteria**	Forty-five knee studies in 45 consecutive symptomatic patients (mean age 54.6 ¬± 20.3 years; 16 males/29 females; 21 right/24 left)	Not stated.	Not listed.

	performed at 3 T in our institution between April 2020 and July 2020 were included.		
**Exclusion criteria**	Cases after ligament reconstruction were excluded.	Not stated.	Not listed.
**MRI machine used to acquire data (magnetic field + vendor)**	3T Siemens with 8-channel knee coil	Not listed.	Not listed.
**MRI sequences used**	2D-FSE T1-weighted (T1WI) and intermediate-weighted images, with and without fat suppression	T1-sagittal, T2-sagittal, T2-coronal, and T2-transverse	3D DESS, IW TSE, and MPR
**MRI images used (i.e. axial, sagittal, 3D volumes)**	Sagittal only	T1-sagittal, T2-sagittal, T2-coronal, and T2-transverse	Coronal and sagittal
**Image reconstruction**	Not listed.	Not listed.	Not listed.
**Machine learning type**	2D CNN	3D CNN	CNNs
**Coding interface used in data processing**	Python	PyTorch	Not listed.
**(Convolutional) Neural Network used**	Original	Original	3D-ResCNN
**Number of data for training**	1799 (81.7%)	70 cases	Not listed.
**Data augmentation used**	Yes	Other: Not used.	Yes
**If yes, what type of data augmentation technique was used?**	We augmented the image data by randomly zooming in and out, rotating within a range of ‚Äì0.15 and +0.15 radians, and flipping it left and right [19]. We primarily focused on fine structures in the knee joints, so we applied such data augmentation.	N/A	Pseudo-labeling approach
**Was a validation performed**	Yes	Yes	Other: No - required in further studies.
**If yes, number of data for validation**	99 (4.5%)	10 cases	N/A
**Internal or external validation**	Internal	External	N/A
**Definition of ground truth reference standard (i.e. supervised learning by radiologist)**	Radiologist or clinician opinion	Not listed.	Not listed.
**Statistical tests run**	Kappa values, To evaluate our DL model, 5 metrics of predictive power (accuracy, average precision, average recall, F-measure, and sensitivity) were calculated on a Neural Network Console ver. 2.1 deep learning library (Sony). In addition, area under the receiver operator characteristic curve (AUROC) values were also calculated with commercial software (SPSS for Windows ver. 28.0, IBM, Armonk, NY, USA).	AUC	p values, accuracy, AUC etc.
**Outcomes measures (metrics of model performance i.e. ROC, performance, sensitivity, specificity, Dice coefficient)**	The accuracy, average precision, average recall, F-measure, sensitivity, and area under the receiver operator characteristic curve (AUROC) of DL for each abnormality were calculated.	AUC	Accuracy, AUC
**Specificity Value**	0.894		
**AUC-ROC Value**	0.931	0.949	0.932
**Accuracy Value**	0.895		0.904
**Sensitivity Value**	0.905		
**Notes/Comments**	No transfer learning listed. Performance metrics as listed for classification of knee abnormalities. Suggests that a study evaluating the diagnostic performance of human	No transfer learning listed. Results are based on an average of the model performance across two datasets. Clinical reason for lack of implementation is	No transfer learning listed. Performance metric is determined by the highest perofrming model that utilised various slices, and a SSL approach to training

	readers when assisted by DL model is required.	listed as problems with interpratibility of features assessed by the CNN.	data. There is no comment on clinical applications.

**Covidence #**	1154	1119	1085
**Study ID**	Rahouma 2021	Couteaux 2019	Roblot 2019
**Title**	Knee Images Classification using Transfer Learning	Automatic knee meniscus tear detection and orientation classification with Mask-RCNN	Artificial intelligence to diagnose meniscus tears on MRI
**Reviewer Name**	Consensus	Consensus	Consensus
**Title**	Knee Images Classification using Transfer Learning	Automatic knee meniscus tear detection and orientation classification with Mask-RCNN	Artificial intelligence to diagnose meniscus tears on MRI
**First author**	Kamel Rahouma	V. Couteaux	V. Roblot
**Year**	2021	2019	2019
**Country in which the study conducted**	Other: Egypt	Other: France	Other: France
**Study design**	Retrospective cohort study	Other: Diagnostic test accuracy study	Retrospective cohort study
**Disease (topic of the study)**	General Abnormalities	Meniscal Pathology	Meniscal Pathology
**Aim of study**	To use transfer learning techniques pplied in training a CNN to classify images of knee to their respective classes.	Classifying MR images of the knee with respect to the presence of tears in the knee menisci, on meniscal tear location, and meniscal tear orientation.	To build and evaluate a high-performance algorithm to detect and characterize the presence of a meniscus tear on magnetic resonance imaging examination (MRI) of the knee
**Follow up**	Other: Not listed.	Other: Not listed	Other: Not listed.
**FDA/TGA/EC mark approved**	No.	No.	No.
**Data source (local or open-source register)**	Other: Stanford ML	Other: Data provided as part of an academic challenge.	Local
**Population description**	Not listed.	1128 images<br><br>The first batch contained 55/257 (21.4%) images with horizontal posterior tears, 46/257 (17.9%) with vertical posterior tears, 13/257 (5.1%) with horizontal anterior tears and 8/257 (3.1%) with vertical anterior tears.<br><br>The second batch contained 107/871 (12.3%) images with horizontal posterior tears, 60/871 (6.9%) with vertical posterior tears, 8/871 (0.9%) with horizontal anterior tears and 3/871 (0.3%) with vertical anterior tears.	700 test set images
**Inclusion criteria**	Not listed.	Not listed.	Not listed.
**Exclusion criteria**	Not listed.	Not listed.	Not listed.
**MRI machine used to acquire data (magnetic field + vendor)**	Not listed.	Not listed.	Not listed.
**MRI sequences used**	Not listed.	Not listed.	T2-weighted
**MRI images used (i.e. axial, sagittal, 3D volumes)**	Sagittal, Coronal, Axial	Sagittal	Sagittal
**Image reconstruction**	Not listed.	Not listed.	Not listed.
**Machine learning type**	Transfer learning - Deep learning-based Convolutional Neural Network (CNN)	Mask region-based convolutional neural network (R-CNN)	Algorithm based on fast-region convolutional neural network (CNN) and faster-region CNN
**Coding interface used in data processing**	Not listed.	Not listed.	Not listed.
**(Convolutional) Neural Network used**	NASNet Mobile	ResNet	Original

**Number of data for training**	Not listed.	1128 total<br>Batch 1 - 257<br>Batch 2 - 871	1123 MRI images
**Data augmentation used**	Other: No.	Other: Not listed.	Yes
**If yes, what type of data augmentation technique was used?**	N/A	N/A	Vertically-flipped version of images
**Was a validation performed**	Other: Not explicitly listed - validation accuracy was calculated but no other information available.	Yes	Yes
**If yes, number of data for validation**	Not listed.	54	700
**Internal or external validation**	Not listed - assumed internal.	Internal	External
**Definition of ground truth reference standard (i.e. supervised learning by radiologist)**	Not listed.	Training labels, reports, or annotations	Training labels, reports, or annotations
**Statistical tests run**	Training and validation accuracy/loss	AUC, accuracy, precision, recall (67%), probability predictions	AUC
**Outcomes measures (metrics of model performance i.e. ROC, performance, sensitivity, specificity, Dice coefficient)**	Classification Accuracy	AUC, accuracy, precision, recall (sensitivity)	AUC
**Specificity Value**			
**AUC-ROC Value**		0.906	0.900
**Accuracy Value**	0.880	0.830	
**Sensitivity Value**		0.670	
**Notes/Comments**	Transfer learning used with NASNet Results taken as an average across all pathologies. Mobile on the ImageNet database. There is no comment on clinical applications.	No transfer learning listed. This study was done as part of a challenge thus limitations in validation or ground truth applications. There is no comment on clinical applications.	No transfer learning listed. Results as listed. Suggests that the lack of data means that the model isn't up to clinical standards yet.

**Covidence #**	920	496	477
**Study ID**	Norman 2018	Iqbal 2020	Haddadian 2022
**Title**	Artificial intelligence pipeline for meniscus segmentation and lesion detection	Deep learning-based automated detection of human knee joint's synovial fluid from magnetic resonance images with transfer learning	Transfer Learning and Data Augmentation in the Diagnosis of Knee MRI
**Reviewer Name**	Consensus	Consensus	Consensus
**Title**	Artificial Intelligence Pipeline For Meniscus Segmentation and Lesion Detection	Deep learning-based automated detection of human knee joint's synovial fluid from magnetic resonance images with transfer learning	Transfer Learning and Data Augmentation in the Diagnosis of Knee MRI
**First author**	B.D. Norman	Imran Iqbal	John Haddadian
**Year**	2018	2020	2022
**Country in which the study conducted**	United States	Other: China	Australia
**Study design**	Retrospective cohort study	Retrospective cohort study	Retrospective cohort study
**Disease (topic of the study)**	Meniscal Pathology	Synovial Fluid Detection	General Abnormalities
**Aim of study**	To use deep learning models to (1) identify the region around the meniscus and then using that<br>region (2) to predict if a lesion is present and if so, its severity.	To apply the deep learning model to detect the synovial fluid of human knee joint from magnetic resonance images.	To investigate the effect of structural variations and various transfer learning implementations on the performance of a deep neural network in the classification task of knee MRI.
**Follow up**	Other: Not listed.	Other: Not listed.	Other: Not listed.
**FDA/TGA/EC mark approved**	No.	No.	No.

**Data source (local or open-source register)**	Other: 3 Previous studies	Local	Other: Stanford dataset
**Population description**	1478 knee MRI subjects with and without OA and after ACL injury were collected from three previous studies (age ¬º 42.79 ¬± 14.75 year, BMI ¬º 24.28 ¬± 3.22 Kg/m2, 48/52 male/female split).	Training set - 49 subjects (mean age 41.7 years; 28 female subjects) from 2015 to 2017 at Shanghai Key Laboratory of Orthopaedic Implants with 1433 images. 890 images (296 transverse, 296 coronal, and 298 sagittal planes) have synovial fluid and 543 images (213 transverse, 151 coronal, and 179 sagittal planes) do not have synovial fluid.<br><br>Development/Test set - 40 subjects (mean age 45.5 years; 17 female subjects) from PC Hospital Liaoning with 15 for development and 25 for testing.	1250 MRIs with 120 exams excluded for use in competition validation.<br><br>1104 (80.6%) of the MRI examinations are labelled as abnormal, 319 (23.3%) have ACL lesions, and 508 (37.1%) have meniscal tears.
**Inclusion criteria**	Not listed.	Not listed.	Not listed.
**Exclusion criteria**	Not listed.	Not listed.	Not listed.
**MRI machine used to acquire data (magnetic field + vendor)**	3T GE Scanner	Not listed.	Not listed.
**MRI sequences used**	3D fast spin-echo (FSE) CUBE sequence	Training - T2 and PD pulse sequences<br><br>Development/Test - proton-density weighting with fat suppression (PDFS), PDFS, and T2 weighting with fat suppression (T2FS).	T2-weighted
**MRI images used (i.e. axial, sagittal, 3D volumes)**	3D volumes	Training - Coronal, Sagittal, Axial for each sequence.<br><br>Development/Test - Coronal proton-density weighting with fat suppression (PDFS), sagittal PDFS, and transverse T2 weighting with fat suppression (T2FS).	Axial, Sagittal, Coronal
**Image reconstruction**	Not listed.	Not listed.	Not listed.
**Machine learning type**	Deep learning - deconvolutional neural network architecture	Deep learning - Transfer learning - CNN	Modified MRNet CNN
**Coding interface used in data processing**	Not listed.	Tensorflow	Pytorch
**(Convolutional) Neural Network used**	Original	Original	AlexNet
**Number of data for training**	3843 (65% of 5912 mVOIs)	1433 images from 49 subjects	1130
**Data augmentation used**	Other: Not listed.	Yes	Yes
**If yes, what type of data augmentation technique was used?**	N/A	Horizontal flip, crop image, crop to aspect ratio, pixel value scale, rotation, adjust brightness, adjust contrast, adjust hue, adjust saturation, distort colour and black patches	Rotation, Shear Mapping, Scale- Li, and in some, colour modification techniques such as adjusting the colour jitter, brightness, and contrast of the image.
**Was a validation performed**	Yes	Yes	Yes
**If yes, number of data for validation**	1182 (20% of 5912 mVOIs)	Not listed.	120
**Internal or external validation**	Internal	Internal	Internal
**Definition of ground truth reference standard (i.e. supervised learning by radiologist)**	Radiologist or clinician opinion	Radiologist or clinician opinion	Training labels, reports, or annotations

**Statistical tests run**	Specificity, sensitivity, ROC, accuracy, confusion matrix	Classification loss, total loss, training time, localisation loss, false positives, false negatives, sensitivity, specificity, precision, F-score, accuracy, dice coefficient, detection time	AUC
**Outcomes measures (metrics of model performance i.e. ROC, performance, sensitivity, specificity, Dice coefficient)**	Specificity, sensitivity, ROC, accuracy	Sensitivity, specificity, precision, F-scores/dice coefficient, accuracy, detection time (approx. 0.71 seconds).	AUC
**Specificity Value**	0.898	0.821	
**AUC-ROC Value**	0.890		0.938
**Accuracy Value**	0.784	0.868	
**Sensitivity Value**	0.820	0.893	
**Notes/Comments**	No transfer learning listed. Results as listed for highest performing model as listed and accuracy is taken as an average across all grades of injury. No comment on clinical applications.	Transfer learning used with the COCO dataset. Results reported as listed. There is no comment on clinical applications.	Transfer learning with ImageNet was utilised in this study. Performance metric is determined by the AUC for the higher performing model (axial view only with colour augmentation) across all pathologies and slices. There is no comment on clinical applications.

**Covidence #**	469	438	412
**Study ID**	Gokay 2022	Shin 2022	Shin 2022
**Title**	Diagnosing Knee Injuries from MRI with Transformer Based Deep Learning	Development of convolutional neural network model for diagnosing tear of anterior cruciate ligament using only one knee magnetic resonance image.	Development of convolutional neural network model for diagnosing meniscus tear using magnetic resonance image.
**Reviewer Name**	Consensus	Consensus	Consensus
**Title**	Diagnosing Knee Injuries from MRI with Transformer Based Deep Learning	Development of convolutional neural network model for diagnosing tear of anterior cruciate ligament using only one knee magnetic resonance image	Development of convolutional neural network model for diagnosing meniscus tear using magnetic resonance image
**First author**	G√∂kay Sezen	Hyunkwang Shin	Hyunkwang Shin
**Year**	2022	2022	2022
**Country in which the study conducted**	Other: Turkey	Other: Korea	Other: Korea
**Study design**	Retrospective cohort study	Retrospective cohort study	Retrospective cohort study
**Disease (topic of the study)**	General Abnormalities	ACL Pathology	Meniscal Pathology
**Aim of study**	To propose a combination of convolution neural network and sequential network deep learning models for detecting general anomalies, ACL tears, and meniscal tears on knee MRI.	To develop a convolutional neural network (CNN) to diagnose anterior cruciate ligament (ACL) tear using only one knee magnetic resonance image of each patient	To develop a CNN to detect meniscal tears and classify tear types using coronal and sagittal magnetic resonance (MR) images of each patient.
**Follow up**	Other: Not listed.	Other: Not listed.	Other: Not listed.
**FDA/TGA/EC mark approved**	No.	No.	No.
**Data source (local or open-source register)**	Other: MRNet dataset	Local	Local
**Population description**	The dataset includes;<br>,Äì 1,104 (80.6%) abnormal exams<br>,Äì 319 (23.3%) ACL (anterior cruciate ligament) tears<br>,Äì 508 (37.1%) meniscal tears	164 patients who had knee injury and underwent knee MRI evaluation at our university hospital from January 2010 to December 2020 (mean age, 43.6 ¬± 17.5 M 108:56). Among the 164 patients, 83 patients,Äô ACLs were torn (20 patients, partial tear; 63 patients, complete tear), whereas 81	599 cases with meniscal tears (medial meniscus tear=384, lateral meniscus tear=167, medial and lateral meniscus tears=48) and 449 cases with-out meniscal tears were used.

		patients,Äô ACLs were intact.	
**Inclusion criteria**	Not listed.	Patients aged ,â• 20 years who had no previous history of knee surgery.	Not listed.
**Exclusion criteria**	Not listed.	Not listed.	Not listed.
**MRI machine used to acquire data (magnetic field + vendor)**	Not listed.	1.5T MR scanner (Phillips Medical Systems, Eindhoven, the Netherlands)	1.5T MR scanner (Philips Medical Systems, Eindhoven, Netherlands)
**MRI sequences used**	Not listed.	Fat-suppressed T2-weighted	Fat-suppressed T2-weighted
**MRI images used (i.e. axial, sagittal, 3D volumes)**	Axial, coronal, and sagittal	Oblique-sagittal	Sagittal & coronal
**Image reconstruction**	Not listed.	Not listed.	Not listed.
**Machine learning type**	Deep learning - convolutional neural network (CNN) and transformer-based networks	Deep learning CNN	Convolutional neural network (CNN)
**Coding interface used in data processing**	Not listed.	Keras using TensorFlow as the backend	PyTorch version 1.7.0
**(Convolutional) Neural Network used**	ResNet	VGG	AlexNet
**Number of data for training**	1130	130 images (79%)	70%
**Data augmentation used**	Yes	Yes	Other: Not listed.
**If yes, what type of data augmentation technique was used?**	Exams were rotated randomly between ,àí25 and 25,ó¶, moved randomly between ,àí25 and 25 pixels, and flipped horizontally with a 50% probability.	Width, zoom, and shear functions were used for data augmentation.	N/A
**Was a validation performed**	Yes	Other: Not listed.	Other: Not listed.
**If yes, number of data for validation**	120	N/A	N/A
**Internal or external validation**	Internal	N/A	Not listed.
**Definition of ground truth reference standard (i.e. supervised learning by radiologist)**	Training labels, reports, or annotations	Radiologist or clinician opinion	Not listed.
**Statistical tests run**	AUC-ROC based on unlisted sensitivity and specificity readings.	Precision, accuracy, recall, AUC	Accuracy, precision, recall, sensitivity, specificity, and area under the curve (AUC)
**Outcomes measures (metrics of model performance i.e. ROC, performance, sensitivity, specificity, Dice coefficient)**	AUC-ROC	AUC, accuracy, precision	Accuracy, precision, recall (42.86%-68.75%), sensitivity, specificity, and area under the curve (AUC).
**Specificity Value**			0.933
**AUC-ROC Value**	0.880	0.941	0.924
**Accuracy Value**		0.941	0.920
**Sensitivity Value**		0.941	0.786
**Notes/Comments**	No transfer learning listed. Performance metric is determined by the average AUC for the higher performing model (ResNet+Transfromer) across all pathologies and slices. There is no comment on clinical applications.	No transfer learning listed. Results as lised.There is no comment on clinical applications.	No transfer learning listed. Results based on detection of the presence of a meniscal tear as listed for medal & lateral tears combined. Potentially lower accuracy may be a reason not clinically implemented but no direct comment.

**Covidence #**	261	248	241
**Study ID**	Liu 2019	Chang 2019	Liu 2018
**Title**	Fully automated diagnosis of anterior cruciate ligament tears on knee mr images by using deep learning	Deep Learning for Detection of Complete Anterior Cruciate Ligament Tear	Deep learning approach for evaluating knee MR images: Achieving high diagnostic performance for cartilage lesion detection
**Reviewer Name**	Consensus	Consensus	Consensus
**Title**	Fully Automated Diagnosis of Anterior Cruciate Ligament	Deep Learning for Detection of Complete Anterior Cruciate Ligament Tear	Deep learning approach for evaluating knee MR images: Achieving high diagnostic

	Tears on Knee MR Images by Using Deep Learning		performance for cartilage lesion detection
**First author**	Fang Liu	Peter D. Chang	Fang Liu
**Year**	2019	2019	2018
**Country in which the study conducted**	United States	United States	United States
**Study design**	Retrospective cohort study	Retrospective cohort study	Retrospective cohort study
**Disease (topic of the study)**	ACL Pathology	ACL Pathology	Osteoarthritis or Cartilage Pathologies
**Aim of study**	To investigate the feasibility of using a deep learning‚Äìbased approach to detect an anterior cruciate ligament (ACL) tear within the knee joint at MRI by using arthroscopy as the reference standard.	To examine the feasibility and incremental benefit of several customized network architectures in evaluation of complete anterior cruciate ligament (ACL) tears.	To determine the feasibility of using a deep learning approach to detect cartilage lesions (including cartilage softening, fibrillation, fissuring, focal defects, diffuse thinning due to cartilage degeneration, and acute cartilage injury) within the knee joint on MR images.
**Follow up**	Other: Not listed.	Other: Not listed.	Other: Not listed.
**FDA/TGA/EC mark approved**	No.	No.	No.
**Data source (local or open-source register)**	Local	Local	Local
**Population description**	350 patients - 175 subjects with a surgically confirmed ACL tear (98 male subjects and 77 female subjects; average age, 27.5 years; age range, 16‚Äì47 years) and 175 subjects with a surgically confirmed intact ACL (100 male subjects and 75 female subjects; average age, 39.4 years; age range, 17‚Äì51 years.	260 patients ages 18‚Äì40. Half of the cases demonstrated a complete ACL tear (624 slices), the other half a normal ACL (3520 slices).	175 patients with knee pain (99 men and 76 women, with an average age of 46.5 years and an age range of 16‚Äì74 years) with imaging from 2010 to 2016.
**Inclusion criteria**	Not listed.	Not listed.	Not listed.
**Exclusion criteria**	Not listed.	Not listed.	Not listed.
**MRI machine used to acquire data (magnetic field + vendor)**	3.0-T imaging unit (Signa ExciteHDx; GE Healthcare, Waukesha, Wis) by using an eight-channel phased-array extremity coil (Invivo, Orlando, Fla)	1.5 & 3T General Electric and Siemens	3.0-T MRI unit (Signa Excite HDx; GE Healthcare, Waukesha, Wis) and eight-channel phased-array extremity coil (Invivo, Orlando, Fla).
**MRI sequences used**	Proton density‚Äìweighted and fat-suppressed T2-weighted fast spin-echo	Proton density (PD) non-fat suppressed	Fat-suppressed T2-weighted fast spin-echo
**MRI images used (i.e. axial, sagittal, 3D volumes)**	Sagittal	Coronal	Sagittal
**Image reconstruction**	Matrix size reconstruction	Not listed.	Matrix size reconstruction.
**Machine learning type**	Deep Learning‚Äìbased ACL Tear Detection System using multiple CNNs	Deep learning - 3D CNN algorithm	Deep learning - CNNs
**Coding interface used in data processing**	TensorFlow (version 1.04; Google, Mountain View, Calif)	Python 3.5 using the open-source TensorFlow r1.2 library	Keras packages (version 2.0.6) with Tensorflow libraries (version 1.0.1)
**(Convolutional) Neural Network used**	DenseNet	ResNet	VGG
**Number of data for training**	200 - 100 with an ACL tear and 100 with an intact ACL	160 (80% of 200)	175 subjects, 1320 patches (random selection of 660 patches with cartilage lesions and 660 patches that show normal cartilage).
**Data augmentation used**	Other: Not listed.	Other: Not listed.	Other: Not listed.
**If yes, what type of data augmentation technique was used?**	N/A	N/A	N/A
**Was a validation performed**	Yes	Yes	Yes
**If yes, number of data for validation**	50 - 25 with ACL tear and 25 with intact ACL	40 (20% of 200)	Not listed - threefold cross-validation information listed as external link.

**Internal or external validation**	Internal	Internal	Internal
**Definition of ground truth reference standard (i.e. supervised learning by radiologist)**	Arthroscopic or surgical findings	Radiologist or clinician opinion	Radiologist or clinician opinion
**Statistical tests run**	Sensitivity, specificity, Receiver operating characteristic (ROC) analysis/area under curve (AUC), two-sided exact binomial tests (using 95% confidence intervals (CIs)).	Sensitivity, specificity, precision, accuracy, positive predictive value (PPV), and negative predictive value (NPV).	ROC, Kappa statistics, sensitivity, specificity, youden index, dice coefficient,
**Outcomes measures (metrics of model performance i.e. ROC, performance, sensitivity, specificity, Dice coefficient)**	Sensitivity, specificity, Receiver operating characteristic (ROC) analysis/area under curve (AUC)	Accuracy, Sensitivity, Specificity, PPV (0.938) & NPV (1.00), AUC	ROC, Kappa statistics (0.76), sensitivity, specificity
**Specificity Value**	0.960	1.000	0.866
**AUC-ROC Value**	0.980	0.971	0.916
**Accuracy Value**			
**Sensitivity Value**	0.960	0.967	0.823
**Notes/Comments**	No transfer learning listed. Results as lised. Compared to radiologist performance. Suggests this study required more validation before clinical implementation.	Transfer learning utilised. Results as listed. Clear comparison against radiologist performance. Further validation required before clinically implemented with a prospective study.	No transfer learning listed. Results are taken as the average between evaluation 1 and 2.There is no comment on clinical applications.

**Covidence #**	229	222	206
**Study ID**	Salmi 2019	Singh 2020	Pedoia 2019
**Title**	A Machine Learning Model for Automation of Ligament Injury Detection Process	Classification of effusion and cartilage erosion affects in osteoarthritis knee MRI images using deep learning model	3D convolutional neural networks for detection and severity staging of meniscus and PFJ cartilage morphological degenerative changes in osteoarthritis and anterior cruciate ligament subjects
**Reviewer Name**	Consensus	Consensus	Consensus
**Title**	A Machine Learning Model for Automation of Ligament Injury Detection Process	Classification of Effusion and Cartilage Erosion Affects in Osteoarthritis Knee MRI Images Using Deep Learning Model	3D Convolutional Neural Networks for Detection and Severity Staging of Meniscus and PFJ Cartilage Morphological Degenerative Changes in Osteoarthritis and Anterior Cruciate Ligament Subjects
**First author**	Cheikh Salmi	Pankaj Pratap Singh	Valentina Pedoia
**Year**	2019	2020	2018
**Country in which the study conducted**	Other: Algeria	Other: India	United States
**Study design**	Retrospective cohort study	Retrospective cohort study	Retrospective cohort study
**Disease (topic of the study)**	ACL Pathology	Osteoarthritis or Cartilage Pathologies	General Abnormalities
**Aim of study**	To generate a model that can extract ACL from MRI input data and classify its different lesions.	To detect and classify OA disease in knee from medical images using deep features.	To evaluate the ability of deep-learning models to detect and stage severity of meniscus and patellofemoral cartilage lesions in osteoarthritis and anterior cruciate ligament (ACL) subjects.
**Follow up**	Other: Not listed.	Other: Not listed.	Other: Not listed.
**FDA/TGA/EC mark approved**	No.	No.	No.
**Data source (local or open-source register)**	Other: Clinical Hospital Centre Rijeka, Croatia dataset	Other: Pascal & COCO datasets collected from the National Institute of Health	Other: Dataset made of 3 previous sets

		(NIH) and also from Invectus Innovation Pvt. Ltd., Noid.	
**Population description**	917 exams - non-injured (690 exams), partially injured (172 exams) and completely torne (55 exams)	349 OA affected knee MRI images	1481 originally with 3 excluded - 1478 knee MRI studies (Approx. 302 unique patients) with and without OA (N = 173), after anterior cruciate ligament (ACL) injury (N = 129) and follow-up post-ACL reconstruction.<br><br>Age = 42.79 ¬± 14.75 year Body mass index [BMI] = 24.28 ¬± 3.22 kg/m2, Gender - 48/52 male/female split
**Inclusion criteria**	Not listed.	Not listed.	Gave informed consent.
**Exclusion criteria**	Not listed.	Not listed.	Poor image quality (3 excluded).
**MRI machine used to acquire data (magnetic field + vendor)**	Not listed.	Not listed.	3T MRI GE (Milwaukee, WI)
**MRI sequences used**	T1-weighted	Not listed.	3D FSE CUBE
**MRI images used (i.e. axial, sagittal, 3D volumes)**	Sagittal	Sagittal	3D volume
**Image reconstruction**	Not listed.	Not listed.	Not listed.
**Machine learning type**	Deep learning - CNNs	Deep learning CNNs - Single shot detection (SSD) model.	Deep learning - 3D Convolutional Neural Network
**Coding interface used in data processing**	TensorFlow	TensorFlow	Native Tensorflow
**(Convolutional) Neural Network used**	Original	VGG	Original
**Number of data for training**	734 exams (80%)	303 images for training	961 (65%)
**Data augmentation used**	Other: Not listed.	Other: Not listed.	Yes
**If yes, what type of data augmentation technique was used?**	N/A	N/A	Random rotation and translation image
**Was a validation performed**	Yes	Yes	Yes
**If yes, number of data for validation**	183 exams (20%)	35 (10%)	296 (20%)
**Internal or external validation**	Internal	Internal	Internal
**Definition of ground truth reference standard (i.e. supervised learning by radiologist)**	Training labels, reports, or annotations	Training labels, reports, or annotations	Radiologist or clinician opinion
**Statistical tests run**	AUC-ROC, sensitivity (sensibility), specificity, precision and associated calculations	IoU, classification loss and localisation loss	Receiver operating characteristic (ROC) curve, specificity and sensitivity, and class accuracy.
**Outcomes measures (metrics of model performance i.e. ROC, performance, sensitivity, specificity, Dice coefficient)**	AUC-ROC, sensitivity, specificity, precision	Not listed.	Receiver operating characteristic (ROC) analysis using specificity and sensitivity and area under the curve (AUC)
**Specificity Value**	0.938		0.811
**AUC-ROC Value**	0.966		0.779
**Accuracy Value**	0.978		
**Sensitivity Value**	0.993		0.849
**Notes/Comments**	No transfer learning listed. Results listed as reported. There is no comment on clinical applications.	Transfer learning utilised. No performance metrics listed. No direct comment on clinical implementation absence. Very focussed on the computer science aspect & coding of the DL model.	No transfer learning listed. Results are listed as the average of all measures for each pathology listed. There is no comment on clinical applications as they believe the study was too prliminary and not ready for clinical implementation.

**Covidence #**	199	186	179
**Study ID**	Hung 2023	Li 2022	Germann 2020
**Title**	Automatic Detection of Meniscus Tears Using Backbone Convolutional Neural Networks on Knee MRI	Identification and diagnosis of meniscus tear by magnetic resonance imaging using a deep learning model	Deep Convolutional Neural Network-Based Diagnosis of Anterior Cruciate Ligament Tears: Performance Comparison of Homogenous Versus Heterogeneous Knee MRI Cohorts with Different Pulse Sequence Protocols and 1.5-T and 3-T Magnetic Field Strengths
**Reviewer Name**	Consensus	Consensus	Consensus
**Title**	Automatic Detection of Meniscus Tears Using Backbone Convolutional Neural Networks on Knee MRI	Identification and diagnosis of meniscus tear by magnetic resonance imaging using a deep learning model	Deep Convolutional Neural Network‚ÄìBased Diagnosis of Anterior Cruciate Ligament Tears: Performance Comparison of Homogenous Versus Heterogeneous Knee MRI Cohorts With Different Pulse Sequence Protocols and 1.5-T and 3-T Magnetic Field Strengths
**First author**	Truong Nguyen Khanh Hung	Jie Li	Christoph Germann
**Year**	2022	2022	2020
**Country in which the study conducted**	Other: Taiwan	Other: China	Other: Switzerland
**Study design**	Retrospective cohort study	Retrospective cohort study	Retrospective cohort study
**Disease (topic of the study)**	Meniscal Pathology	Meniscal Pathology	ACL Pathology
**Aim of study**	To train and evaluate a deep learning model for automated detection of meniscus tears on knee magnetic resonance imaging (MRI).	To improve the diagnostic accuracy and efficiency, a deep learning model was employed in this study and the identification efficiency was evaluated for meniscus tears.	To clinically validate a Deep Convolutional Neural Network (DCNN) for the detection of surgically proven anterior cruciate ligament (ACL) tears in a large patient cohort and to analyze the effect of magnetic resonance examinations from different institutions, varying protocols, and field strengths.
**Follow up**	Other: Not listed.	Other: Not listed.	Other: Not listed.
**FDA/TGA/EC mark approved**	No.	No.	No.
**Data source (local or open-source register)**	Local	Local	Local
**Population description**	The following details only the testing set of data:<br>- 200 patients from Cho Ray hospital:<br><br>Age - 42.53 +/- 2.76<br>BMI (kg/m^2) - 25.73 +/- 2.31<br>Gender - 27 women (24.54%)<br>Intact menisci - 97 (48.5%)<br>Injured menisci - 103 (51.5%)	200 internal, 180 external	512 people - 231 women and 281 men; mean age, 34 ¬± 15 years; range, 10‚Äì77 years
**Inclusion criteria**	Patients who are had arthroscopic knee surgery to confirm meniscus tears between January 1, 2020, and December 31, 2021, at Cho Ray Hospital (Hospital 1) and Hoan My Hospital (Hospital 2).	The diagnostic criteria for a meniscal tear included abnormal meniscal hyperintensity, and hyperintensity involving at least one articular surface of the meniscus or reaching the free edge of the meniscus.<br><br>In the image processing stage, the images without motion artifacts or any other magnetic artifacts were included.	(1) history of knee pain<br>(2) 1.5-T or 3-T MRI of the knee joint performed after the injury, either at our institution or at an outside institution including at least one coronal and sagittal fluid-sensitive fat-suppressed pulse sequence<br>(3) arthroscopic knee surgery performed at our institution by specialized knee surgeons within 4 months after the knee MRI, and

			(4) agreement to participate in the study.
**Exclusion criteria**	Previous knee surgery.	Not listed.	(1) previous knee surgery with metal implants or previous ACL reconstruction<br>(2) presence of an intraarticular or invading neoplasm<br>(3) MR arthrography<br>(4) osseous avulsion of the ACL, and<br>(5) technically insufficient or incomplete examination.
**MRI machine used to acquire data (magnetic field + vendor)**	Either the MAG-NETOM Skyra 3T (Siemens Healthcare, Erlangen, Germany) (at Cho Ray Hospital) or the SIGNA 3T (GE healthcare, Chicago, IL, USA) (at Hoan My Hospital) with both using a dedicated 15-channel transmit/receive knee coil.	3.0 T MR imaging system (United Imaging Co., Ltd., Shanghai, China) with a dedicated knee coil.	Local Institution - 1.5-T or 3-T MRI systems (Magnetom Avanto fit or Magnetom Skyra fit, Siemens Healthcare, Erlangen, Germany) with dedicated 15-channel transmit/receive knee coils.<br>External Institutions (58) - 1.5-T or 3-T MRI scanners of 4 different vendors (Canon Medical Systems Otawara, Japan; GE Healthcare, Waukesha, WI; Philips Healthcare, Best, the Netherlands; and Siemens Healthcare, Erlangen, Germany).
**MRI sequences used**	T1-weighted proton density (PD) fast spin-echo (FSE) with fat saturation and T2-weighted FSE with fat saturation sequences.	Fat-suppressed proton density-weighted (PDW) & fat-suppressed T2	Fluid-sensitive fat-suppressed turbo and fast spin echo
**MRI images used (i.e. axial, sagittal, 3D volumes)**	Coronal & Sagittal	Sagittal	Coronal and sagittal
**Image reconstruction**	Not listed.	Not listed.	Not listed.
**Machine learning type**	Deep learning based model - CNN - You Only Look Once architecture (YOLOv4)	Deep Learning network structure - Mask regional convolutional neural network (R‚ÄìCNN)	Deep learning - Deep Convolutional Neural Network (DCNN)
**Coding interface used in data processing**	Not listed.	Not listed.	Keras (2.2.4) framework with TensorFlow (1.11) backend
**(Convolutional) Neural Network used**	DarkNet	ResNet	Original
**Number of data for training**	234	504 (220 patients)	4802
**Data augmentation used**	Other: Not listed.	Yes	Other: Not listed.
**If yes, what type of data augmentation technique was used?**	N/A	Geometric transformation (horizontal, vertical and diagonal mirroring), lighting/brightness adjustment, Gaussian filtering and noise addition (such as salt and pepper noise) were used to expand the number of samples in the dataset.	N/A
**Was a validation performed**	Yes	Yes	Yes
**If yes, number of data for validation**	270 total<br>- 150 (internal)<br>- 120 (external)	220 internal, 180 external	500
**Internal or external validation**	Internal (Hoan Myy Hospital) & External (MRNet)	Internal & external	Internal
**Definition of ground truth reference standard (i.e. supervised learning by radiologist)**	Arthroscopic or surgical findings	Training labels, reports, or annotations	Multiple ie. Arthroscopic or surgical findings & Training labels, reports, or annotations & Radiologist or clinician opinion
**Statistical tests run**	Sensitivity, specificity, prevalence, positive predictive	Diagnostic accuracy, IoU, average precision (AP),	T test, sensitivity, specificity, Fisher exact, McNemar test,

	value, negative predictive value, accuracy, and receiver operating characteristic curve were used to evaluate the performance of the detection model. Two-way analysis of variance (ANOVA), Wilcoxon signed-rank test, 95% confidence intervals (CIs) and Tukey,Äôs multiple tests were used to evaluate differences in performance between the model and radiologists.	recall, loss function<br><br>Note about AP - AP50 and AP75 were APs when IoU threshold was greater than 0.5 and greater than 0.75, respectively. APs, APm, and API were represented as the AP for small objects (area <322), medium objects (322<area <962), and large objects (962<area), respectively.	receiver operating characteristic (ROC) curve analyses with the calculation of the area under the curve (AUC) and 95% confidence interval (CI), kappa statistics
**Outcomes measures (metrics of model performance i.e. ROC, performance, sensitivity, specificity, Dice coefficient)**	Sensitivity, specificity, accuracy, and the area under the receiver operating characteristic (ROC) curve (AUC).	Precision, sensitivity & accuracy	AUC ROC, Sensitivity, Specificity, F1 score
**Specificity Value**	0.920		0.931
**AUC-ROC Value**			0.935
**Accuracy Value**	0.958	0.864	
**Sensitivity Value**	0.985	0.838	0.961
**Notes/Comments**	No transfer learning listed. Internal validation results listed as is for performance measures as the highest result. There is no comment on clinical applications.	No transfer learning listed. Results are based on Box ResNet50 model with an average taken across healthy, torn & degenerative menisci. Lack of clinical implementation may be due to the lack of homogenised standard datasets across different people and different MRI equipment.	No transfer learning listed. There is no comment on clinical applications.

**Covidence #**	178	177	151
**Study ID**	Gupta 2022	Xie 2021	Joshi 2022
**Title**	Intelligent detection of knee injury in MRI exam	Deep Learning-Based MRI in Diagnosis of Fracture of Tibial Plateau Combined with Meniscus Injury	Anterior Cruciate Ligament Tear Detection Based on Deep Convolutional Neural Network
**Reviewer Name**	Consensus	Consensus	Consensus
**Title**	Intelligent detection of knee injury in MRI exam	Deep Learning-Based MRI in Diagnosis of Fracture of Tibial Plateau Combined with Meniscus Injury	Anterior Cruciate Ligament Tear Detection Based on Deep Convolutional Neural Network
**First author**	Sanjana Gupta	Xiaoxiao Xi	Kavita Joshi
**Year**	2022	2021	2022
**Country in which the study conducted**	Other: United Arab Emirates	Other: China	Other: India
**Study design**	Retrospective cohort study	Retrospective cohort study	Retrospective cohort study
**Disease (topic of the study)**	General Abnormalities	Tibial Plateau Fracture with Meniscus Injury	ACL Pathology
**Aim of study**	To solve and go through the problem of Knee injuries detection in medical diagnosis and solve the problem by processing MRI by building a multi-model convolutional neural network (CNN) consisting of four pre-trained models,ÄîVGG16, VGG19, ResNet152V2, InceptionV3, DenseNet201 to help classify knee injuries from MRI scans into ACL tears, meniscal tears or abnormalities in the knee.	To explore the application value of magnetic resonance imaging (MRI) images based on deep learning algorithms in the diagnosis of tibial plateau fractures combined with meniscus injuries.	To improve feature distinctiveness, thus improving the representation capability of the complex knee MRI texture for ACL tear detection using knee MRI images.
**Follow up**	Other: Not listed.	Other: Not listed. Suggested future potential to have follow up with "an expanded sample size... to strengthen the findings of the study"	Other
**FDA/TGA/EC mark approved**	No.	No.	No.

**Data source (local or open-source register)**	Other: MRNet dataset	Local	Other: MRNet dataset
**Population description**	1,104 (80.6%) abnormal exams, with 319 (23.3%) ACL tears and 508 (37.1%) meniscal tears. No other population data was provided.	80 patients diagnosed with tibial plateau fractures. 48 male patients and 32 female patients, aged between 24 and 67 years.<br><br>Thee tibial plateau fractures in 44 patients arose from traffic trauma, and the remaining 36 patients fell to have tibial plateau fractures.<br><br>According to the 6-classification method proposed by Schatzker in 1974 based on fracture X-rays, the subjects were divided into 6 types: 8 cases of type I, 12 cases of type II, 16 cases of type III, 4 cases of IV, 8 cases of type V, and 32 cases of type VI.	1295 patients - 845 normal and 450 abnormal samples to form the dataset. 30% was used for testing (approx. 389). Demographics were not listed.
**Inclusion criteria**	Not listed.	(I) patients with tibial plateau fractures combined with meniscus injury;<br>(II) aged 16,Äì70 years;<br>(III) lateral plateau fractures showed significant displacement or collapse, and the medial plateau articular surface remained relatively intact;<br>(IV) patients not having the over-knee surgery; and<br>(V) patients without surgical contraindications	Not listed.
**Exclusion criteria**	Not listed.	(I) patients with other knee joint diseases and severe knee joint deformities;<br>(II) patients with coagulation dysfunction or those unable to have the surgery for other reasons; and<br>(III) patients who did not sign the surgical consent form.<br><br>Note - None of the above patients who participated in this study had undergone knee surgery and had undergone MRI and intraoperative meniscus examination during the treatment.	Not listed.
**MRI machine used to acquire data (magnetic field + vendor)**	Not listed.	Not listed	Not listed.
**MRI sequences used**	Not listed.	Not listed	T2
**MRI images used (i.e. axial, sagittal, 3D volumes)**	Axial, coronal, and sagittal.	Not listed (only sagittal images shown in figures).	Sagittal
**Image reconstruction**	Not listed.	Regridding image reconstruction algorithm.	Not listed.
**Machine learning type**	Deep learning - Multi-model convolutional neural network (CNN)	Deep learning convolutional neural network (CNN)	Deep convolutional neural network (DCNN)
**Coding interface used in data processing**	Not listed.	Not listed	Python-OpenCV programming
**(Convolutional) Neural Network used**	Multiple - VGG16, VGG19, ResNet152V2, InceptionV3, DenseNet201	Original	Original
**Number of data for training**	1130 exams (1088 patients)	Not listed	70% of the data were used for training (approx. 907)

**Data augmentation used**	Yes	Other: Not listed	Other: No - future recommendations in the paper suggest using an augmented dataset.
**If yes, what type of data augmentation technique was used?**	Not listed.	Not listed	N/A
**Was a validation performed**	Other: Not listed.	Other: Not listed	Yes
**If yes, number of data for validation**	N/A	Not listed	Not listed.
**Internal or external validation**	N/A	Not listed	Not listed.
**Definition of ground truth reference standard (i.e. supervised learning by radiologist)**	Training labels, reports, or annotations	Arthroscopic or surgical findings	Not listed.
**Statistical tests run**	Accuracy and F1 scores	Sensitivity, Specificity and Accuracy, sample t tests, four-point position, chi-square test.	Accuracy, recall rate, precision, and the F1 score
**Outcomes measures (metrics of model performance i.e. ROC, performance, sensitivity, specificity, Dice coefficient)**	Accuracy and F1 scores	Sensitivity, Specificity and Accuracy	Accuracy, recall rate, precision, and the F1 score
**Specificity Value**		0.932	
**AUC-ROC Value**			
**Accuracy Value**	0.875	0.953	0.966
**Sensitivity Value**		0.969	0.967
**Notes/Comments**	No transfer learning listed. Performance metric is determined by the best performing model (VGG19). There is no comment on clinical applications.	No transfer learning listed. Results as listed. There is no comment on clinical applications.	No transfer learning listed. Model performance based on CPDCNN-Adam which has the highest performance. Compared against other DL model perofrmance in other studies. There is no comment on clinical applications.

**Covidence #**	148	144	142
**Study ID**	Kara 2021	Jeon 2021	Zhang 2023
**Title**	Detection and Classification of Knee Injuries from MR Images Using the MRNet Dataset with Progressively Operating Deep Learning Methods	Interpretable and Lightweight 3-D Deep Learning Model for Automated ACL Diagnosis	Multi-level classification of knee cartilage lesion in multimodal MRI based on deep learning
**Reviewer Name**	Consensus	Consensus	Consensus
**Title**	Detection and Classification of Knee Injuries from MR Images Using the MRNet Dataset with Progressively Operating Deep Learning Methods	Interpretable and Lightweight 3-D Deep Learning Model for Automated ACL Diagnosis	Multi-level classification of knee cartilage lesion in multimodal MRI based on deep learning
**First author**	Ali Can Kara	Young Seok Jeon	Lirong Zhang
**Year**	2021	2021	2023
**Country in which the study conducted**	Other: Turkey	Other: Singapore (Primary)	Other: China
**Study design**	Retrospective cohort study	Retrospective cohort study	Retrospective cohort study
**Disease (topic of the study)**	Meniscal Pathology	ACL Pathology	Osteoarthritis or Cartilage Pathologies
**Aim of study**	To build progressively operating deep learning models that could detect meniscus injuries, anterior cruciate ligament (ACL) tears and knee abnormalities in magnetic resonance imaging (MRI).	To propose an interpretable and lightweight 3D deep neural network model that diagnoses anterior cruciate ligament (ACL) tears from a knee MRI exam.	To propose a multi-classification model of knee cartilage injury based on deep learning.
**Follow up**	Other: Not listed.	Other: Not listed - suggested will be in future in "we will investigate the accuracy of	Other: Not listed.

		our model using multiple MRI vendors and different magnetic field machines."	
**FDA/TGA/EC mark approved**	No.	No.	No.
**Data source (local or open-source register)**	Other: MRNet dataset	Other: Chiba & Stanford datasets	Local
**Population description**	No demographic information was provided.<br><br>There are 1104 abnormal, 319 ACLs, and 508 menisci in the dataset.	Patient demographics were suggested to be found in the original dataset study.	209 patients admitted for KC lesion and arthroscopic surgery, aged between 18 and 55. 51 cases of arthroscopic cartilage lesion level I, 54 of level II, 52 of level III, and 52 of level IV. And 57 healthy volunteers (no knee discomfort and surgical history) are selected.
**Inclusion criteria**	Not listed.	Not listed.	Not listed.
**Exclusion criteria**	Noisy and/or damaged images from the dataset at the Model 1 stage since they were unsuitable for diagnosis.	Not listed.	Not listed.
**MRI machine used to acquire data (magnetic field + vendor)**	Not listed.	1. Chiba dataset - A 3.0 T MRI (Ingenia CX, Philips Medical Systems) with 16 channel transmit/receive knee coil, and a 1.5 T MRI (Excelart Vantage, Canon Medical Systems) with 7 channel transmit/receive knee coil<br>2. Stanford dataset GE scanners (GE Discovery, GE Healthcare, Waukesha, WI) with standard knee MRI coil were used for the extraction.	Not listed.
**MRI sequences used**	Not listed.	1. Chiba dataset - non-fat suppressed sequence<br>2. Stanford dataset - fat-suppressed sequence	T1WI, T2 mapping, and PDWI-FS
**MRI images used (i.e. axial, sagittal, 3D volumes)**	Coronal, sagittal and axial	Not listed.	Sagittal
**Image reconstruction**	Not listed.	Not listed.	Not listed.
**Machine learning type**	Deep learning CNN - integrating the convolutional neural networks (CNN) and the denoising autoencoder models	3D Convolutional Neural Network (CNN)	Deep learning - CNNs
**Coding interface used in data processing**	Not listed.	PyTorch	Not listed.
**(Convolutional) Neural Network used**	3D-ResCNN	Original	AlexNet
**Number of data for training**	1130	Not listed.	146 patients - 70%
**Data augmentation used**	Other: No	Yes	Other: Not listed.
**If yes, what type of data augmentation technique was used?**	N/A	3D affine transform and random volume cropping.	N/A
**Was a validation performed**	Yes	Yes	Other: Not listed.
**If yes, number of data for validation**	120	Not listed.	Not listed.
**Internal or external validation**	Internal	Internal<br><br>The performance evaluation on the Chiba dataset was executed with 5-fold cross-validation.<br><br>The Stanford dataset‚Äôs performance evaluation is executed on the validation	Not listed.

		set since the test set is sequestered.	
**Definition of ground truth reference standard (i.e. supervised learning by radiologist)**	Training labels, reports, or annotations	Not listed.	Not listed.
**Statistical tests run**	Loss values, Accuracy, sensitivity, specificity, Matthew‚Äôs correlation coefficient (MCC) and the area under the receiver operating characteristic curve (ROC-AUC) values	As below.	Accuracy, F1 scores, recall, precision and associated calculations
**Outcomes measures (metrics of model performance i.e. ROC, performance, sensitivity, specificity, Dice coefficient)**	Accuracy, sensitivity, specificity, Matthew‚Äôs correlation coefficient (MCC) (0.3801-0.6702) and the area under the receiver operating characteristic curve (ROC-AUC)	ROC-AUC, Sensitivity & specificity<br><br>Note - the Chiba dataset included +/- std for all readings however the Stanford dataset did not.	Accuracy, F1 scores, recall (0.9933-1.0000), precision
**Specificity Value**		0.975	
**AUC-ROC Value**	0.932	0.983	
**Accuracy Value**	0.904		0.997
**Sensitivity Value**		0.930	0.997
**Notes/Comments**	Transfer learning applied from Imagenet. Results based on model best performance with multi-view scans, GAP & residuals. There is no comment on clinical applications.	No transfer learning listed. Performance metrics taken from highest performing DL model (OUR+Conv on the Chiba dataset). Suggested reason for lack of clinical implementation is inconsistencies or variation in image quality in MRI unlike mammography and the wide range of pathologies that can be detected.	Transfer learning/pre-training done with AlexNet. Recall averaged across all levels. Accuracy reported as presented. There is no comment on clinical applications.

**Covidence #**	140	134	104
**Study ID**	Truong 2021	Huo 2022	Hu 2022
**Title**	Prediction of anterior cruciate ligament injury from MRI using deep learning	Automatic Grading Assessments for Knee MRI Cartilage Defects via Self-ensembling Semi-supervised Learning with Dual-Consistency	Deep Learning-Based Multimodal 3 T MRI for the Diagnosis of Knee Osteoarthritis
**Reviewer Name**	Consensus	Consensus	Consensus
**Title**	Prediction of anterior cruciate ligament injury from MRI using deep learning	Automatic Grading Assessments for Knee MRI Cartilage Defects via Self-ensembling Semi-supervised Learning with Dual-Consistency	Deep Learning-Based Multimodal 3T MRI for the Diagnosis of Knee Osteoarthritis
**First author**	Nguyen Khanh Hung Truong	Jiayu Huo	Yong Hu
**Year**	2021	2022	2022
**Country in which the study conducted**	Other: Taiwan & Vietnam	Other: China	Other: China
**Study design**	Retrospective cohort study	Retrospective cohort study	Retrospective cohort study
**Disease (topic of the study)**	ACL Pathology	Osteoarthritis or Cartilage Pathologies	Osteoarthritis or Cartilage Pathologies
**Aim of study**	To focus on a comprehensive high accurate prediction of ACL injury based on MRI medical images, and also demonstrate the ability of AI in practical and outline conceptual prediction and diagnosis frameworks for other types of knee injuries in the future.	To propose a semi-supervised frame-work to effectively use unlabeled data for better evaluation of knee cartilage defect grading.	To investigate the application effect of deep learning model combined with different magnetic resonance imaging (MRI) sequences in the evaluation of cartilage injury of knee osteoarthritis (KOA).
**Follow up**	Other: Not listed.	Other: Not listed.	Other: Not listed - "The follow-up research should focus on this problem to

			strengthen the findings of the study."
**FDA/TGA/EC mark approved**	No.	No.	No.
**Data source (local or open-source register)**	Local	Local	Local
**Population description**	799 patients<br><br>- 622 ACL injuries<br>- 137 normal knees	1296 knee scans from Shanghai Jiao Tong University Affiliated Sixth People‚Äôs Hospital for the knee cartilage defect assessment task from 2011-2017.	104 patients with KOA who were admitted to the hospital between October 20, 2018, and February 20, 2021. No history of high intensity exercise training.<br><br>Gender - 35 female, 69 male<br>Age - 27 <30 years old, 67 30-45 years old, 10 >45 years old<br>BMI - 20 <18.5kg/m^2, 63 18.5-23.9kg/m^2, 21 >23.9kg/m^2<br>Location - 58 left knee, 46 right knee
**Inclusion criteria**	Knee MRI examinations from January 1st, 2015 ‚Äì December 31st, 2019.	Not listed.	1) no past or recent history of major knee trauma<br>(2) no previous history of infectious diseases<br>(3) no previous surgical history<br>(4) no previous use of drugs affecting cartilage<br>(5) patients with complete clinical data, and<br>(6) no contraindications for MRI examination.
**Exclusion criteria**	History of previous knee surgery.	Not listed.	(1) poor MRI image quality<br>(2) patients without arthroscopic examination information<br>(3) patients with congenital or acquired knee deformity, and<br>(4) body mass index (BMI) is too high or too low.
**MRI machine used to acquire data (magnetic field + vendor)**	Not listed.	Philips Achieva 3.0T TX MRI scanner (Philips Healthcare, Best, Netherlands) with eight surface coils	3.0 T superconducting magnetic resonance imaging system was used, with 15 channel phased array surface coil. Vendor was not listed.
**MRI sequences used**	Not listed.	T2-weighted fat-suppressed	Proton density-weighted inhibition (PDWI-FS), 3D double-echo stable water excitation (3D-DESS-WE), T2 Mapping, T2* Mapping, T1 Mapping
**MRI images used (i.e. axial, sagittal, 3D volumes)**	Coronal, axial, sagittal	Sagittal	Sagittal 3D-Dess-WE<br>T2 mapping (plane not listed)<br>T2‚àó mapping (plane not listed)<br>T1 mapping (plane not listed)
**Image reconstruction**	Not listed.	Matrix resizing to 256x256	SRCNN algorithm, SDD algorithm, EDSR algorithm and MSRN (model used in the study).
**Machine learning type**	Not listed.	Semi-supervised deep learning - dual-consistency mean teacher (DC-MT) method ie. a two stage framework involving the DCT and the aggregation network/single 2D slice classification network.	Deep learning - image superresolution algorithm based on an improved multiscale wide residual network model

**Coding interface used in data processing**	Not listed.	Pytorch	Not listed.
**(Convolutional) Neural Network used**	DenseNet	SE-ResNeXt50	MRSN
**Number of data for training**	Not listed - only states "small".	Not listed.<br><br>Only thing listed is - sample 10 labeled images and 30 un-labeled images for each mini-batch, following the ratio setting of labeled and unlabeled data in the MT framework in reference to training.	Not listed.
**Data augmentation used**	Other: Not listed.	Yes	Other: Not listed.
**If yes, what type of data augmentation technique was used?**	N/A	Random brightness, random contrast, and random horizontal flip	N/A
**Was a validation performed**	Other: Not listed.	Yes	Other: Not listed.
**If yes, number of data for validation**	N/A	Not listed - five-fold cross-validation on the whole dataset.	N/A
**Internal or external validation**	N/A	Internal	N/A
**Definition of ground truth reference standard (i.e. supervised learning by radiologist)**	Training labels, reports, or annotations	Radiologist or clinician opinion	Arthroscopic or surgical findings
**Statistical tests run**	Precision, accuracy, sensitivity & associated calculations.	Area under the roc curve (AUC), F1-score, accuracy (ACC), sensitivity (SEN), and specificity (SPE), Detected region ratio (IoR)	Peak signal noise ratio (PSNR), structural similarity (SSIM), one way analysis of variance (ANOVA), diagnostic accuracy, sensitivity, specificity, kappa consistency test value
**Outcomes measures (metrics of model performance i.e. ROC, performance, sensitivity, specificity, Dice coefficient)**	Precision, accuracy, sensitivity	AUC-ROC, F1-score, accuracy, sensitivity and specificity	Accuracy rate and kappa consistency test value
**Specificity Value**		0.903	
**AUC-ROC Value**		0.903	
**Accuracy Value**	0.792	0.873	0.847
**Sensitivity Value**	0.974	0.796	
**Notes/Comments**	No transfer learning listed. Performance metrics taken as an average across three planes of imaging. Lack of clinical implementation likely due to low detection system due to lack of data with various vendors and protocols.	No transfer learning listed. Results taken from best model performance including 100% labelled data. No comment on clinical applications but suggests that performance can be improved.	No transfer learning listed. Performance metric is based on model performance using 3D-DS-WE which had the highest performance and was taken as an average across all grades of tears. There is no comment on clinical applications and suggest further study be conducted to confirm findings.

**Covidence #**	103	101	96
**Study ID**	Minamoto 2022	Shakhovska 2022	Siouras 2022
**Title**	Automated detection of anterior cruciate ligament tears using a deep convolutional neural network	Comparative Analysis of Backbone Networks for Deep Knee MRI Classification Models	Automated Recognition of healthy Anterior Cruciate Ligament in Sagittal MR images using Lightweight Deep Learning
**Reviewer Name**	Consensus	Consensus	Consensus
**Title**	Automated detection of anterior cruciate ligament tears using a deep convolutional neural network	Comparative Analysis of Backbone Networks for Deep Knee MRI Classification Models	Automated Recognition of healthy Anterior Cruciate Ligament in Sagittal MR images using Lightweight Deep Learning
**First author**	Yusuke Minamoto	Nataliya Shakhovska	Athanasios Siouras

**Year**	2022	2022	2022
**Country in which the study conducted**	Other: Japan	Other: Ukraine	Other: Greece
**Study design**	Retrospective cohort study	Other: Comparative Analysis	Retrospective cohort study
**Disease (topic of the study)**	ACL Pathology	General Abnormalities	ACL Pathology
**Aim of study**	To evaluate the accuracy of a CNN system in diagnosing ACL ruptures by a single slice from a knee MRI and to compare the results with that of experienced human readers.	To compare different types of feature extraction networks for the same classification task, in terms of accuracy and performance.	To develop a robust and lightweight deep learning pipeline for identifying ACL in 3D MRI data of healthy knees.
**Follow up**	Other: Not listed.	Other: Not listed.	Other: Not listed.
**FDA/TGA/EC mark approved**	No.	No.	No.
**Data source (local or open-source register)**	Local	Other: MRNet dataset	Other: MRNet dataset
**Population description**	One hundred MR images from 93 consecutive patients with an ACL injury (mean age 27.2¬±10.6 years, 46 images in 45 males and 54 images in 48 females) and 100 MR images from 100 consecutive patients with an intact ACL (mean age 26.1¬±11.9 years, 67 images in 67 males and 33 images in 33 females) were obtained (Table 1). Seven patients in the ACL-injured group had two MRI scans in the pre-surgical period mainly due to a delay between the time of surgery and the initial injury.	1370 knee MRI exams performed at Stanford University Medical Center. The dataset contains 1104 (80.6%) abnormal exams, with 319 (23.3%) ACL tears and 508 (37.1%) meniscal tears.	1,370 knee MRI tests performed at the Stanford University Medical Center.
**Inclusion criteria**	Patients with and without ACL injury, in which the diagnosis was confirmed by arthroscopy.	Not listed.	Knees that were entirely healthy (n=266) for training and testing. A sample (n=319) of knees with ACL rupture was also investigated to evaluate the capacity of our methodology on injured knees.
**Exclusion criteria**	Patients who did not receive pre-operative imaging by either a 1.5 T (T) or a 3.0 T scanner.	Not listed.	Not listed.
**MRI machine used to acquire data (magnetic field + vendor)**	1.5 T or 3.0 T MRI scanner (vendor not listed).	1.5T (43.4%) and 3T (56.6%) GE scanners (GE Discovery, GE Healthcare, Waukesha, WI, USA) with a standard knee MRI coil	Not listed.
**MRI sequences used**	Proton density-weighted	T1 weighted, T2 with fat saturation, proton density (PD) weighted, T2 with fat saturation, and PD weighted with fat saturation.	Not listed - assumed 3D DESS.
**MRI images used (i.e. axial, sagittal, 3D volumes)**	Sagittal	Axial, coronal, and sagittal:<br><br>Coronal T1 weighted, Coronal T2 with fat saturation, Sagittal proton density (PD) weighted, sagittal T2 with fat saturation, and Axial PD weighted with fat saturation.	Sagittal
**Image reconstruction**	Not listed.	Not listed.	Not listed.
**Machine learning type**	Deep learning - CNN	MRNet architecture	Deep learning - object detection network (CNN)
**Coding interface used in data processing**	Tensorflow, version 1.12.0	Not listed.	Pytorch
**(Convolutional) Neural Network used**	Xception	Multiple - AlexNet, VGG11, VGG16, Resnet, Efficientnet	DarkNet

**Number of data for training**	80	Not listed. Assumed to be 1370 exams (entire MRNet database)	186 (70%)
**Data augmentation used**	Yes	Yes	Yes
**If yes, what type of data augmentation technique was used?**	Random rotation between -20 and 20 degrees, a width and height shift range of 0.2 each, and a random horizontal flip.	Horizontal flip, horizontal flip and cropping, or random affine	Adjustments to image brightness (between -32% and +32%), hue (between -39¬∞ and +39¬∞), exposure (between -30% and +30%), blurring (up to 1.75px), and random noise (up to 7% of pixels) addition.
**Was a validation performed**	Yes	Yes	Yes
**If yes, number of data for validation**	20	Not listed.	53 (20%)
**Internal or external validation**	Internal	Not listed. Assumed external - MRNet validation dataset.	Internal
**Definition of ground truth reference standard (i.e. supervised learning by radiologist)**	Radiologist or clinician opinion	Training labels, reports, or annotations	Radiologist or clinician opinion
**Statistical tests run**	True-positive, true-negative, false-positive, and false-negative rate, ROC, AUC, sensitivity, specificity, accuracy, Youden index, McNemar test.	Area Under the Receiver Operating Characteristics Curve (ROC-AUC), accuracy, f1 score, and Cohen,Äôs Kappa, confusion matrix and associated calculations.	IoU, true positive, false positives, mean average precision (mAP) score
**Outcomes measures (metrics of model performance i.e. ROC, performance, sensitivity, specificity, Dice coefficient)**	Sensitivity, specificity, accuracy	Area Under the Receiver Operating Characteristics Curve (ROC-AUC), accuracy, f1 score, and Cohen,Äôs Kappa.	mean average precision (mAP) score
**Specificity Value**	0.860		
**AUC-ROC Value**	0.942	0.855	
**Accuracy Value**	0.885	0.799	
**Sensitivity Value**	0.910		
**Notes/Comments**	No transfer learning listed. Results as reported. Lack of clinical application likely due to lack of clinical information and lower diagnostic performance but no direct comment.	Pre trained/trnafser learning with AlexNet. Performance based on VGG16 which reported the highest results in this study. The AUC ROC is an average taken across all planes and pathologies listed. There is no comment on clinical applications.	Transfer learning utilised. Different performance metrics used. There is no comment on clinical applications or reasons for lack of clinical deployment.

**Covidence #**	83	81	76
**Study ID**	Azcona 2020	Pandey 2021	Rizk 2021
**Title**	A Comparative Study of Existing and New Deep Learning Methods for Detecting Knee Injuries using the MRNet Dataset	Deep Convolutional Neural Network-Based Knee Injury Classification Using Magnetic Resonance Imaging	Meniscal lesion detection and characterization in adult knee MRI: A deep learning model approach with external validation
**Reviewer Name**	Consensus	Consensus	Consensus
**Title**	A Comparative Study of Existing and New Deep Learning Methods for Detecting Knee Injuries using the MRNet Dataset	Deep Convolutional Neural Network Based Knee Injury Classification Using Magnetic Resonance Imaging	Meniscal lesion detection and characterization in adult knee MRI: A deep learning model approach with external validation
**First author**	David Azcona	Rishi Pandey	B. Rizk
**Year**	2020	2020	2021
**Country in which the study conducted**	Other: Ireland	Other: India	Other: Switzerland
**Study design**	Other: Comparative study	Retrospective cohort study	Retrospective cohort study
**Disease (topic of the study)**	General Abnormalities	General Abnormalities	Meniscal Pathology
**Aim of study**	Present a comparative study of existing and new techniques to detect knee injuries by	To develop a predictive model capable of automatically determining whether an injury is	To evaluate a deep learning approach for the detection of meniscal tears and their characterization

	leveraging Stanford‚Äôs MRNet Dataset.	abnormal, ACL tear or meniscal tear.	(presence/absence of migrated meniscal fragment).
**Follow up**	Other: Not listed.	Other: Not listed.	Other: Not listed.
**FDA/TGA/EC mark approved**	No.	No.	No.
**Data source (local or open-source register)**	Other: MRNet dataset	Other: MRNet dataset	Local
**Population description**	1370 knee MRI exams with:<br>i) 1,104 (80.6%) abnormal exams<br>ii) 319 (23.3%) ACL (anterior cruciate ligament) tears<br>iii) 508 (37.1%) meniscal tears	1370 knee MRI data categorizing into three classes abnormal, ACL tear and meniscal tear obtained from 1370 patients.	8058 examinations with 7903 patients. The population consisted in 48.1% of female and 51.9% of male patients, with a mean age of 44.8 years (range 16‚Äì89) and a mean weight of 74.3 kg (range 38‚Äì186).
**Inclusion criteria**	Not listed.	Not listed.	Knee MRI examinations between 2009<br>and 2018 from 11 medical imaging centers in Switzerland
**Exclusion criteria**	Not listed.	Not listed.	Patients under the age of 16 (N = 309) and those with a known past knee surgical history (N = 2189) were excluded
**MRI machine used to acquire data (magnetic field + vendor)**	Not listed.	Not listed.	13 MRI scanners including<br><br>54% (4348) 1T Philips Panorama<br>36.3% (2929) 3T Philips Ingenia<br>4.9% (392) 1.5T GE ONI MSK Extreme<br>4.1% (330) 1.5T GE Optima MR430s<br>0.7% (53) 3T GE Signa Pioneer<br>0% (4)1.5T GE Signa HDxt<br>0% (2) 3T SIEMENS Skyra
**MRI sequences used**	Not lised.	Not listed.	Proton density (PD) fat suppressed (FS)-weighted images
**MRI images used (i.e. axial, sagittal, 3D volumes)**	3D volumes, or Axial, sagittal, coronal	Sagittal, axial, coronal	Coronal & Sagittal
**Image reconstruction**	Not listed.	Not listed.	Not listed.
**Machine learning type**	Deep learning models including:<br>1. Deep Residual Network with Transfer Learning (2D)<br>2. Deep Residual Network from Scratch & Use a Fixed Number of Slices (2D)<br>3. Multi-Plane Deep Residual Network (3D)<br>4. Multi-Plane Multi-Objective Deep Residual Network (3D)	Transfer learning - convolutional neural networks	Deep learning model based on CNNs
**Coding interface used in data processing**	Javascript	Tensorflow and Keras open source neural network libraries	Python 3.6, Keras 2.2.5, Tensorflow 1.15.0, Scikit-learn 0.22.1, and Numpy 1.19.1
**(Convolutional) Neural Network used**	ResNet	VGG	Original
**Number of data for training**	1. Not listed.<br>2. 15 interpolated or 17 standard slices<br>3. 45 images (15 slices/plane)<br>4. 45 images (15 slices/plane)	1096 images (80%)	6221
**Data augmentation used**	Yes	Other: Not listed.	Other: No.
**If yes, what type of data augmentation technique was used?**	1. horizontal flip, random contrast, random gamma, random brightness, contrast limited adaptive histogram	N/A	N/A

	equalisation, sharpen, emboss & overlay, random brightness contrast, centre crop (height & width are 150), random crop (height & width are 150).<br>2. Same as 1 but the study removed the transformations that needed three channels: Random Brightness, Contrast Limited Adaptive Histogram Equalisation and Random Brightness Contrast.<br>3. Not listed.<br>4. Not listed.		
**Was a validation performed**	Yes	Yes	Yes
**If yes, number of data for validation**	Not listed.	Not explicitly listed - 274 (assumed same as test set based on validation accuracy calculations)	Internal - 1538 internal for the tear detection model<br><br>External - 226 ie. 20% of 1130 of the MRNet dataset
**Internal or external validation**	Internal	Internal - not explicitly listed.	Internal & External
**Definition of ground truth reference standard (i.e. supervised learning by radiologist)**	Training labels, reports, or annotations	Not listed.	Radiologist or clinician opinion
**Statistical tests run**	Loss function, validation AUC	Validation accuracy, accuracy, false positive rate, true positive rate, ROC	IoU values and their associated standard deviations, AUC, sensitivity, specificity, accuracy values as well as their respective confidence intervals.
**Outcomes measures (metrics of model performance i.e. ROC, performance, sensitivity, specificity, Dice coefficient)**	Validation AUC	Accuracy, ROC	AUC, sensitivity, specificity and accuracy
**Specificity Value**			0.860
**AUC-ROC Value**	0.934		0.885
**Accuracy Value**		0.775	0.845
**Sensitivity Value**			0.780
**Notes/Comments**	Transfer learning applied from Imagenet. Reported AUC is the average AUC across all pathologies for DL models that analysed axial, coronal and sagittal slices seperately in a combined model.	Transfer learning with ImageNet was utilised in this study.VGG16 was reported as performance in this study even through VGG19 was also explored as an average across all pathologies. There is no comment on clinical applications and lack of clinical implementation.	No transfer learning listed. Reuslts based on detection of meniscus tear and averaged between the medial & lateral menisci. Lack of clinical implementation attributed to the need for further work to be dome to cover broader structures analysis of knee components in a structured and standardized way.

**Covidence #**	65	51	49
**Study ID**	Tack 2021	Sridhar 2022	Zhang 2020
**Title**	A Multi-Task Deep Learning Method for Detection of Meniscal Tears in MRI Data from the Osteoarthritis Initiative Database	A Torn ACL Mapping in Knee MRI Images Using Deep Convolution Neural Network with Inception-v3	Deep Learning Approach for Anterior Cruciate Ligament Lesion Detection: Evaluation of Diagnostic Performance Using Arthroscopy as the Reference Standard
**Reviewer Name**	Consensus	Consensus	Consensus
**Title**	A Multi-Task Deep Learning Method for Detection of Meniscal Tears in MRI Data from the Osteoarthritis Initiative Database	A Torn ACL Mapping in Knee MRI Images Using Deep Convolution Neural Network with Inception-v3	Deep Learning Approach for Anterior Cruciate Ligament Lesion Detection: Evaluation of Diagnostic Performance Using Arthroscopy as the Reference Standard
**First author**	Alexander Tack	S. Sridha	Lingyan Zhang
**Year**	2021	2022	2020

**Country in which the study conducted**	Other: Germany	Other: India	Other: China
**Study design**	Retrospective cohort study	Retrospective cohort study	Retrospective cohort study
**Disease (topic of the study)**	Meniscal Pathology	ACL Pathology	ACL Pathology
**Aim of study**	To develop a method that provides an efficient, robust and automated way to detect and better locate meniscal tears in MRI data, that is, the detection of tears with respect to the anatomical regions in which they occur.	To propose a Deep Convolution Neural Network (DCNN) based Inception-v3 deep transfer learning (DTL) model for classifying the ACL tear MRI images.	To determine the feasibility of using a deep learning approach to detect ACL injuries within the knee joint on MRI.
**Follow up**	Other: Not listed.	Other: Not listed.	Other: Not listed.
**FDA/TGA/EC mark approved**	No.	No.	No.
**Data source (local or open-source register)**	Other: Osteoarthritis Initiative database	Other: MRNet dataset	Local
**Population description**	DESS: 2399<br>- Age - 61.88+/-8.87<br>- Sex - 1489 female, 910 male<br>- BMI (kg/m^2) - 29.01 +/- 4.79<br><br>IW TSE: 2396<br>- Age - 61.89+/-8.86<br>- Sex - 1487 female, 909 male<br>- BMI (kg/m^2) - 29.08 +/- 4.79	The MRNet dataset includes 1,370 knee MRI images diagnosed at Stanford University Medical Center.)ere are 1,104 (80.6%) abnormal images, 319 (23.3%) ACL tears, and 508 (37.1%) meniscal tears in this dataset	Finally, 408 subjects, aged 16,Äì80 years, were enrolled, including 163 (39.9%) patients with ACL injury (including complete rupture) and 245 patients without ACL injury.<br><br>Age - 40.9 +/- 16.0<br>Gender - Female 40.2%, Male 59.8%
**Inclusion criteria**	Not listed.	Not listed.	- Imaged from October 2011 to March 2019<br>- Underwent arthroscopic surgery and had corresponding MRI images at the site.
**Exclusion criteria**	Not listed.	Not listed.	Previous ACL surgery, severe OA, poor SNR, motion artefacts.
**MRI machine used to acquire data (magnetic field + vendor)**	3T Siemens Trio	1.5T (43.4%) and 3T (56.6%) GE scanners (GE Discovery, GE Healthcare, Waukesha, and WI) were used for examination with a regular knee MRI coil.	1.5T Achieva (302 patients) or 3.0T Ingenia (106 patients) with an 8-channel knee coil (Philips Healthcare, Best, The Netherlands).
**MRI sequences used**	Double Echo Steady-State (DESS) and Intermediate-Weighted Turbo Spin-Echo (IW TSE)	T1 weighted, proton density (PD) weighted, PD weighted with fat saturation, T2 with fat saturation, and T2 with fat saturation	2D PDW-SPAIR
**MRI images used (i.e. axial, sagittal, 3D volumes)**	3D volumes (DESS) and sagittal (IW TSE)	Coronal, sagittal, axial:<br><br>- Coronal T1 weighted<br>- Sagittal proton density (PD) weighted<br>- Axial PD weighted with fat saturation<br>- Coronal T2 with fat saturation<br>- Sagittal T2 with fat saturation	Sagittal - 3D volumes
**Image reconstruction**	Not listed.	Not listed.	3D image reconstruction
**Machine learning type**	3D Convolutional Neural Network (CNN)	Transfer Learning - Deep Convolution Neural Network (DCNN)	Deep learning - Classification CNN
**Coding interface used in data processing**	PyTorch 1.9	Not listed.	Pytorch
**(Convolutional) Neural Network used**	ResNet	Original	DenseNet
**Number of data for training**	2397 - 50% of all data<br><br>(1) DESS - 1200<br>(2) IW TSE - 1197	959 images (70%)	285
**Data augmentation used**	Yes	Other: Not listed.	Yes

**If yes, what type of data augmentation technique was used?**	In training, random cropping around the RoI using the dice similarity coefficient, horizontal flips, rotations, Gaussian noise, and intensity scaling are applied with 50% probability.<br><br>For the Full-scale approach, we perform random cropping of up to 10% along coronal, 20% sagittal and 20% axial direction.<br><br>In the BB-crop approach, random crops are performed by uniformly cropping within a 20% margin around the menisci.<br><br>The BB-loss approach uniformly samples possible crops around the menisci. All cropped images are resampled with trilinear interpolation to attain consistent sizes per approach and dataset.	N/A	Randomly flipped each volume, shifted ¬±10 voxels and rotated ¬±10 in the x and y coordinates.
**Was a validation performed**	Yes	Yes	Yes
**If yes, number of data for validation**	1718 - 15% of all data<br><br>(1) DESS - 1359<br>(2) IW TSE - 359	411 images (30%)	81
**Internal or external validation**	Internal	Internal	Internal
**Definition of ground truth reference standard (i.e. supervised learning by radiologist)**	Not listed.	Training labels, reports, or annotations	Arthroscopic or surgical findings
**Statistical tests run**	IoU, sensitivity, specificity, ROC-AUC	True positive, true negative, false positive, and false negative, accuracy, precision, recall, specificity, and F-measure	Classification accuracy, sensitivity, specificity, positive predictive value (PPV), negative predictive value (NPV), and area under the receiver operating characteristic curve (AUC).
**Outcomes measures (metrics of model performance i.e. ROC, performance, sensitivity, specificity, Dice coefficient)**	AUC	Accuracy, precision, recall, specificity, and F-measure	Accuracy, sensitivity, specificity, PPV (0.940), NPV (0.976), AUROC
**Specificity Value**		0.963	0.944
**AUC-ROC Value**	0.935		0.960
**Accuracy Value**		0.954	0.957
**Sensitivity Value**		0.951	0.976
**Notes/Comments**	No transfer learning utilised. Results based on 3D data which performed higher than 2D data as an average between the lateral and medial meniscus for all aspects (anterior horn, body, posterior horn). ResNet50 BB-loss model employed. No direct comment on clinical implementation.<br><br>1. ResNet50 encoder with an MLP classifier head<br>2. Resnet-C-26 encoder with an MLP classifier head<br>2. ResNet50 encoder with two (2) MLP classifier heads	Pre trained/transfer learning with Inception-v3.Results provided as is for the model. There is no comment on clinical applications ie. no reason why it is not implemented.	No transfer learning listed. Results based on an average taken from the highest performing model (5-fold cross validation with various inputs).There is no comment on clinical applications ie. no reason why it is not implemented. Discussed better performance than radiologists.

Covidence #	38	37	35
Study ID	Fritz 2020	Astuto 2021	Li 2022
Title	Deep convolutional neural network-based detection of meniscus tears: comparison with radiologists and surgery as standard of reference	Automatic deep learning‚Äìassisted detection and grading of abnormalities in knee MRI studies	Automated meniscus segmentation and tear detection of knee MRI with a 3D mask-RCNN
Reviewer Name	Consensus	Consensus	Consensus
Title	Deep convolutional neural network-based detection of meniscus tears: comparison with radiologists and surgery as standard of reference	Automatic Deep Learning‚Äìassisted Detection and Grading of Abnormalities in Knee MRI Studies	Automated meniscus segmentation and tear detection of knee MRI with a 3D mask-RCNN
First author	Benjamin Fritz	Bruno Astuto	Yuan‚ÄëZhe Li and Yi Wang
Year	2020	2021	2022
Country in which the study conducted	Other: Switzerland	United States	Other: China
Study design	Retrospective cohort study	Retrospective cohort study	Retrospective cohort study
Disease (topic of the study)	Meniscal Pathology	General Abnormalities	Meniscal Pathology
Aim of study	To clinically validate a fully automated deep convolutional neural network (DCNN) for detection of surgically proven meniscus tears.	To test the hypothesis that artificial intelligence (AI) techniques can aid in identifying and assessing lesion severity in the cartilage, bone marrow, meniscus, and anterior cruciate ligament (ACL) in the knee, improving overall MRI inter-reader agreement.	To present a fully automatic 3D deep convolutional neural network (DCNN) for meniscus segmentation and detects arthroscopically proven meniscus tears
Follow up	Other: Not listed.	Other	Other: Not listed.
FDA/TGA/EC mark approved	No.	No.	No.
Data source (local or open-source register)	Local	Other: Collected within three previous studies (from 2011 to 2014) under the National Institutes of Health‚ÄìNational Institute of Arthritis and Musculoskeletal and Skin Diseases grants P50AR060752 and R01AR046905 from the University of California, San Francisco (San Francisco, Calif), the Hospital for Special Surgery (New York City, NY), and the Mayo Clinic (Rochester, Minn).	Local
Population description	The study population consisted of 46 women and 54 men with a mean age of 39.9 years (standard deviation (SD) 14.3 years; range 14‚Äì74 years). Age was not significantly different between women (mean 40.1 ¬± 14.2 years) and men (mean 39.7 ¬± 14.6 years) with p = 0.893. Sixty-four patients were examined on a 1.5 Tesla (T) and 36 patients were examined on a 3T MR scanner	294 patients with a mean age of 43 years +/- 15; body mass index, 24.28 kg/m^2 +/- 3.22; and 52% (153) women.	546 knees with meniscal injuries were recorded from 533 patients admitted to the Department of Orthopedics of the second Affiliated Hospital of Fujian Medical University. All patients had arthroscopically confirmed meniscus injury and underwent MRI before arthroscopy of the affected knee. Among 533 patients, 336 were males, and 197 were females, ranging in age from 9 to 78 years, with an average of 51.3¬±10.5 years. Knees with meniscus injury were divided into meniscus tear and non-tear of the meniscus. Among 546 knees with meniscus injuries, 331 knees with meniscus tears and 215 knees with non-tears of meniscus were found.

**Inclusion criteria**	(1) MRI of the knee joint performed at our institution on a clinical 1.5 Tesla or 3 Tesla clinical whole-body MRI system using our standard protocols for evaluation of knee pain<br>(2) arthroscopic knee surgery performed at our institution by a specialized knee surgeon, at a time interval of less than 3 months after the knee MRI; and<br>(3) signed informed consent for retrospective data analysis.	Study 1/3 - at least 35 years old.<br>Study 2&3/3 - Post ACL injury or single bundle ACLR by board-certified, fellowship-trained orthopedic surgeon within 3 years (n = 61 and n = 64).	Meniscus that has been diagnosed arthroscopically and have been scanned with an MRI.
**Exclusion criteria**	Patients were excluded in case of previous knee surgery or impaired image quality due to motion.	Study 1/3 - concurrent use of an investigational drug, fracture or surgical intervention in the study knee, and any contraindications to MRI (n = 169).<br>Study 2&3/3 - Not using soft-tissue grafts; for the hamstrings, allografts, autografts, or posterior tibialis allografts.	Unavailable or incomplete clinical or MRI information; poor quality MRI images with a low signal-to-noise ratio (SNR)
**MRI machine used to acquire data (magnetic field + vendor)**	1.5 T or 3 T MRI system (Magnetom Avanto fit or Magnetom Skyra fit, Siemens Healthcare, Erlangen, Germany) with a dedicated 15 channel transmit/receive knee coil.	3T GE Discovery 750HD MRI scanners (GE Healthcare) with eight surface coils.	3.0T Philips with a dedicated 15-channel transmitting/receiving knee joint coil
**MRI sequences used**	Fluid sensitive fat supressed eg. short-tau inversion or intermediate weighted (IW) sequences	High-resolution 3D fast-spin-echo CUBE sequence	Proton density weighted (PDW)
**MRI images used (i.e. axial, sagittal, 3D volumes)**	Coronal & Sagittal	3D volume	Sagittal
**Image reconstruction**	Not listed.	Not listed.	To reconstruct the entire sagittal PDW sequence for knee MRI for use with the 3D convolution kernels in the network, patches from the test set were then applied to the model, and the resulting reconstruction was then reassembled.
**Machine learning type**	Deep learning - Deep convolutional neural network (DCNN)	Deep learning - Three-dimensional convolutional neural networks	Deep learning - Deep Convolution Neural Network (DCNN)
**Coding interface used in data processing**	Keras framework on the TensorFlow backend	Not listed.	Not listed.
**(Convolutional) Neural Network used**	Original	V-Net	ResNet
**Number of data for training**	18,520	70% of 1435 images (1004-1005 images)	382 (70%)
**Data augmentation used**	Other: Not listed.	Yes	Other: Not listed.
**If yes, what type of data augmentation technique was used?**	N/A	All models were trained using data augmentation. Details on training parameters, the split strategy, and the augmentation strategy are described in Appendix E2 (supplement).	N/A
**Was a validation performed**	Yes	Yes	Yes
**If yes, number of data for validation**	1000	15% of 1435 (215-216 images)	164 (30%)
**Internal or external validation**	Internal	External	Internal

**Definition of ground truth reference standard (i.e. supervised learning by radiologist)**	Arthroscopic or surgical findings AND Training labels, reports, or annotations	Radiologist or clinician opinion	Arthroscopic or surgical findings
**Statistical tests run**	t test, sensitivity, specificity, accuracy, McNemar test, receiver operating characteristic (ROC) curve analyses with calculation of the area under the ROC curves (AUC), Cohen‚Äôs kappa.	Sample t tests, linear-weighted Cohen k, etc.	Dice accuracy, sensitivity, specificity, FROC, receiver operating characteristic (ROC) curve analysis, and bootstrap test statistics.
**Outcomes measures (metrics of model performance i.e. ROC, performance, sensitivity, specificity, Dice coefficient)**	Sensitivity, Specificity, Accuracy, F1 score & AUC-ROC	ROC-AUC, sensitivity, specificity	Dice accuracy, sensitivity, specificity, FROC, receiver operating characteristic (ROC) curve analysis
**Specificity Value**	0.900	0.890	0.785
**AUC-ROC Value**	0.961	0.930	0.907
**Accuracy Value**	0.850		0.924
**Sensitivity Value**	0.710	0.850	0.941
**Notes/Comments**	No transfer learning listed. Results based on an average taken from the highest performing model (final model + radiomic features) across intact, partially ton and fully ruptured reported data.There is no comment on clinical applications. Discussed better performance than radiologists & use as an assistive tool but wihtout reason.	No transfer learning listed. Reason not in clinics is heavoly realted to the lack of validation and data availability.	No transfer learning listed. Results based on an average taken from the highest performing model.There is no comment on clinical applications.

**Covidence #**	32	30	26
**Study ID**	Namiri 2020	Zhu 2022	Dung 2023
**Title**	Deep learning for hierarchical severity staging of anterior cruciate ligament injuries from mri	Fully RNN for Knee Ligament Tear Classification and Localization in MRI Scans	End-to-end deep learning model for segmentation and severity staging of anterior cruciate ligament injuries from MRI
**Reviewer Name**	Consensus	Consensus	Consensus
**Title**	Deep Learning for Hierarchical Severity Staging of Anterior Cruciate Ligament Injuries from MRI	Fully RNN for Knee Ligament Tear Classification and Localization in MRI Scans	End-to-end deep learning model for segmentation and severity staging of anterior cruciate ligament injuries from MRI
**First author**	Nikan K. Namiri	Kaiyue Zhu	Nguyen Tan Dung
**Year**	2020	2022	2023
**Country in which the study conducted**	United States	United States	Other: Viet Nam
**Study design**	Retrospective cohort study	Retrospective cohort study	Retrospective cohort study
**Disease (topic of the study)**	ACL Pathology	ACL Pathology	ACL Pathology
**Aim of study**	To evaluate the diagnostic utility of two convolutional neural networks (CNNs) for severity staging of anterior cruciate ligament (ACL) injuries	To propose a fully Recurrent Neural Network (RNN) for detecting Anterior Cruciate Ligament (ACL) tears using MRI scans.	To develop a semi-supervised segmentation and classification deep learning model for the diagnosis of anterior cruciate ligament (ACL) tears on MRI based on a semi-supervised framework, double-linear layers U-Net (DCLU-Net).
**Follow up**	Other: Not listed.	Other: Not listed.	Other: Not listed - suggested in future.
**FDA/TGA/EC mark approved**	No.	No.	No.
**Data source (local or open-source register)**	Local	Other: MRNet & kneeMRI data sets (2 datasets are utilised in this study)	Local
**Population description**	1243 knee MR images (1008 intact, 18 partially torn, 77 fully	1. The MRNet dataset - 1,370 knee MRI exams	297 patients

	torn, and 140 reconstructed ACLs) from 224 patients (mean age, 47 years 6 14 [standard deviation]; 54% women).	performed at Stanford University Medical Center. The dataset contains 1,104 (80.6%) abnormal exams, with 319 (23.3%) ACL tears and 508 (37.1%) meniscal tears.<br>2. The kneeMRI Dataset - contains sagittal views with three different ACL diagnoses: not injured (690 cases), partially injured (172 cases), and completely ruptured (55 cases).	Gender - 28% female, 72% male<br>Age - 29-44<br>BMI (km/m^2) - 21.8-29.2<br>ACL Condition - Intact (36%), Partially torn (33%), Fully ruptured (31%)
**Inclusion criteria**	OA group - reported knee pain, aching, or stiffness on most days per month during the past year, reported use of medication for knee pain on most days per month during the past year, or exhibited any possible radiologic sign of knee osteoarthritis (Kellgren-Lawrence grade > 0), and age of 36 years or older.<br><br>Control group - no knee pain or stiffness in either knee and if no use of medications for knee pain in the last year was reported, and if no radiologic evidence of osteoarthritis on either knee was noted (Kellgren-Lawrence grade 0).	Not listed.	Patients who underwent arthroscopic knee surgery between January 2018 and December 2022.
**Exclusion criteria**	Concurrent use of an investigational drug, history of fracture, total knee replacement in the study knee, and any contraindications to MRI.	Not listed.	Previous knee surgery.
**MRI machine used to acquire data (magnetic field + vendor)**	3-T MRI scanners (GE Healthcare, Waukesha, Wis) and eight surface coils.	Not listed.	1.5-T (MAGNETOM Skyra, Siemens Healthcare) with a lower extremity knee coil.
**MRI sequences used**	3D fast spin-echo-Cube proton density,Äìweighted	Not listed.	Fat-saturated (FS) proton density (PD) fast spin-echo sequence (FSE)
**MRI images used (i.e. axial, sagittal, 3D volumes)**	Sagittal oblique - 3D volumes	3D volumes	Sagittal
**Image reconstruction**	Not listed.	Not listed - assumed some form of reconstruction required for 3D volume analysis from sagittal images in kneeMRI dataset.	The input MR images were reconstructed in regard to the matrix size which was changed from 256x232 and 512x512 to 256x256.
**Machine learning type**	Deep learning based convolutional neural networks (CNNs)	Deep learning - Recurrent Neural Network (RNN)	Dual-modal deep learning model - DCLU-Net
**Coding interface used in data processing**	The 3D CNN was developed in TensorFlow (Google, Mountain View, Calif) and the 2D CNN in PyTorch (Facebook, Menlo Park, Calif).	Not listed.	Not listed.
**(Convolutional) Neural Network used**	AlexNet	DarkNet	DCLU-Net
**Number of data for training**	70% - approximately 870 images from 157 patients	1419 frames (Each exam/patient data contains 4-6 frames so 236-355 patients)	247
**Data augmentation used**	Yes	Other: Not listed.	Other: Not listed.
**If yes, what type of data augmentation technique was used?**	Three-dimensional translations and zooming	N/A	N/A
**Was a validation performed**	Yes	Other: Not listed.	Yes

**If yes, number of data for validation**	10% - approximately 124 images from 22 patients	N/A	247
**Internal or external validation**	Internal	N/A	Internal
**Definition of ground truth reference standard (i.e. supervised learning by radiologist)**	Radiologist or clinician opinion	Radiologist or clinician opinion	Training labels, reports, or annotations
**Statistical tests run**	Accuracy, linear-weighted Cohen k, sensitivity, specificity, McNemar test, Fisher exact test, two-sample t tests.	Recall, precision, and F1 score, Average Precision (AP)	Sensitivity, specificity, accuracy and AUROC curve, confusion matrices, root mean square error, t tests and dice coefficient
**Outcomes measures (metrics of model performance i.e. ROC, performance, sensitivity, specificity, Dice coefficient)**	Sensitivity, specificity, weighted Cohen k (0.83), accuracy	Precision, and F1 score	Accuracy, sensitivity, specificity, AUROC, confusion matrices, dice coefficient, root mean square error (0.05-0.06)
**Specificity Value**	0.917		0.8966
**AUC-ROC Value**			
**Accuracy Value**	0.920		0.880
**Sensitivity Value**	0.947		0.820
**Notes/Comments**	Transfer learning/pre-training with ImageNet. The 2D CNN reported higher performance compared to the 3D CNN but the 3D CNN was not pre-trained like the 2D with ImageNet. Sensitivity and specificity was calculated for the 2D CNN as an average across all severities of ACL injury. There is no comment on clinical applications.	No transfer learning listed. All data was reported as F1 scores (no sensitivity, accuracy, AUC etc.). There is no comment on clinical applications.	No transfer learning listed. Results based on an average taken from the medial and lateral meniscus. This article suggests that the model is not advanced enough for clinical practice.

**Covidence #**	22	3
**Study ID**	Awan 2021	Tran 2022
**Title**	Efficient Detection of Knee Anterior Cruciate Ligament from Magnetic Resonance Imaging Using Deep Learning Approach	Deep learning to detect anterior cruciate ligament tear on knee MRI: multi-continental external validation
**Reviewer Name**	Consensus	Consensus
**Title**	Efficient Detection of Knee Anterior Cruciate Ligament from Magnetic Resonance Imaging Using Deep Learning Approach	Deep learning to detect anterior cruciate ligament tear on knee MRI: multi-continental external validation
**First author**	Mazhar Javed Awan	Alexia Tran
**Year**	2021	2021
**Country in which the study conducted**	Other: Study - Malaysia, Pakistan, Iraq & Spain (contributions from each author listed at the bottom from the above locations)<br><br>Dataset - from Croatia	Other: France
**Study design**	Retrospective cohort study	Retrospective cohort study
**Disease (topic of the study)**	ACL Pathology	ACL Pathology
**Aim of study**	To further enhance the automatic performance, without involving a radiologist, by using a deep learning model to detect the anterior cruciate ligament by an inspecting MRI.	To develop a deep-learning algorithm for anterior cruciate ligament (ACL) tear detection and to compare its accuracy using two external datasets.
**Follow up**	Other: Not listed.	Other: Not listed.
**FDA/TGA/EC mark approved**	No.	No.
**Data source (local or open-source register)**	Local	Local
**Population description**	917<br><br>ACL injury status - 690 are healthy, 172 partials and 55 complete ruptured.	The included population consisted of 17,738 patients, 1,744 (9.8%) of which had at least two magnetic resonance (MR) MRI examinations, with an average of 2.2 MR scans. Mean age was 44 years with a standard deviation (SD) of 17 years and a female/male ratio of 48% (8,514) / 52% (9,224).

**Inclusion criteria**	Not listed.	Older than 16 years old.<br>Signed consent form.
**Exclusion criteria**	Not listed.	Younger than 16 years old.
**MRI machine used to acquire data (magnetic field + vendor)**	1.5T Siemens (Muenchen, Germany)	1T-3T Philips Healthcare; GE Healthcare; and Siemens Healthcare MRI machines:<br><br>84% Philips<br>12% GE<br>4% Siemens<br><br>3T - 32%<br>1.5T - 17%<br>1T - 51%
**MRI sequences used**	Proton density-weighted fat suppression	Fat-suppressed proton density or T2-weighted
**MRI images used (i.e. axial, sagittal, 3D volumes)**	Sagittal	Coronal and sagittal
**Image reconstruction**	Not listed.	Not listed.
**Machine learning type**	Deep learning - convolutional neural network (CNN)	Deep convolutional neural network
**Coding interface used in data processing**	Keas (Version 1.0) backend Tensor Flow	Keras deep learning library (keras.io) and a TensorFlow backend were used.
**(Convolutional) Neural Network used**	ResNet	Original
**Number of data for training**	2387 samples (after class balancing) ie. 75% of total data	70%, 13,836 examinations
**Data augmentation used**	Yes	Other: Not listed.
**If yes, what type of data augmentation technique was used?**	1. Set input mean to 0 over the dataset<br>2. Divide inputs by standard deviation of dataset<br>3. Epsilon for Zero-phase whitening (ZCA) whitening<br>4. Set mode for filling points outside the input boundaries<br>5. Randomly flip images horizontally<br>6. Randomly flip images vertically	N/A
**Was a validation performed**	Yes	Yes
**If yes, number of data for validation**	950	Internal - 3,953 examinations (20%)<br>External - 183 exams (20% of 917) and one dataset does not list the validation number.
**Internal or external validation**	Internal - train/test split (random splitting) and K-fold (3 & 5) cross-validation	Internal & External
**Definition of ground truth reference standard (i.e. supervised learning by radiologist)**	Training labels, reports, or annotations	Training labels, reports, or annotations
**Statistical tests run**	Test loss, test accuracy, precision, sensitivity, F1-score, specificity, weighted average, AUC-ROC	Censitivity, specificity, accuracy, and area under the receiver operating characteristic curve (AUC)<br><br>Note - various other statistical tests were run but were often unrelated to the model of interest (comparison data) or testing dataset.
**Outcomes measures (metrics of model performance i.e. ROC, performance, sensitivity, specificity, Dice coefficient)**	Precision, sensitivity, F1-score, specificity, weighted average, AUC-ROC	Receptor operating characteristics (ROC) curves, area under the curve (AUC), sensitivity, specificity, and accuracy.
**Specificity Value**	0.947	0.910
**AUC-ROC Value**	0.980	0.942
**Accuracy Value**	0.920	0.873
**Sensitivity Value**	0.917	0.765
**Notes/Comments**	No transfer learning listed. Performance metric is determined by the top[performing model (Customised ResNet-14 5 Fold Cross-validation). The accuracy rating is only applicable to partial tears as no other accuracy measures were listed for healthy or fully torn ACLs. There is no comment on clinical applications.	No transfer learning listed. Performance metrics are derived from the average of the networks re-trianing on two datasets. Suggest lack of clinical implementation is lack of studies that evaluate the value of AI tools for clinical practice.

Appendix D

A table of the average values for four selected performance metrics (specificity, AUC-ROC, accuracy and sensitivity) recorded against if transfer learning was utilised across all studies. Note that if the performance metric was not listed, it was not included in the average.

Transfer Learning Use	Specificity	AUC-ROC	Accuracy	Sensitivity
**Transfer Learning Used (n=17)**	0.896	0.914	0.871	0.923
**Transfer Learning Not Used (n=36)**	0.903	0.922	0.889	0.875

Appendix E

A table of the average values for four selected performance metrics (specificity, AUC-ROC, accuracy and sensitivity) recorded against the ground truth or reference standard for each study.

Study ID	Ground Truth or Reference Standard	Specificity	AUC-ROC	Accuracy	Sensitivity
Yeoh 2023	Training labels, reports, or annotations		0.945	0.812	
Voinea 2023	Training labels, reports, or annotations		0.965	0.871	
Berrimi 2023	Training labels, reports, or annotations		0.813		
Yeoh 2023	Training labels, reports, or annotations			0.825	0.792
Ying 2023	Arthroscopic or surgical findings AND Training labels, reports, or annotations	0.787	0.772	0.749	0.750
Kasuya 2023	Radiologist or clinician opinion	0.894	0.931	0.895	0.905
Couteaux 2019	Non-clinican or non-specialist opinion		0.906	0.830	0.670
Roblot 2019	Training labels, reports, or annotations		0.900		
Norman 2018	Radiologist or clinician opinion	0.898	0.890	0.784	0.820
Iqbal 2020	Radiologist or clinician opinion	0.821		0.868	0.893
Haddadian 2022	Training labels, reports, or annotations		0.938		
Gokay 2022	Training labels, reports, or annotations		0.880		
Shin 2022	Radiologist or clinician opinion		0.941	0.941	0.941
Liu 2019	Arthroscopic or surgical findings	0.960	0.980		0.960
Chang 2019	Radiologist or clinician opinion	1.000	0.971		0.967
Liu 2018	Radiologist or clinician opinion	0.866	0.916		0.823
Salmi 2019	Training labels, reports, or annotations	0.938	0.966	0.978	0.993
Singh 2020	Training labels, reports, or annotations				
Pedoia 2019	Radiologist or clinician opinion	0.811	0.779		0.849
Hung 2023	Arthroscopic or surgical findings	0.920		0.958	0.985
Li 2022	Training labels, reports, or annotations			0.864	0.838
Germann 2020	Multiple ie. Arthroscopic or surgical findings and Training labels, reports, or annotations and Radiologist or clinician opinion	0.931	0.935		0.961
Gupta 2022	Training labels, reports, or annotations			0.875	
Xie 2021	Arthroscopic or surgical findings	0.932		0.953	0.969
Kara 2021	Training labels, reports, or annotations		0.932	0.904	
Truong 2021	Training labels, reports, or annotations			0.792	0.974
Huo 2022	Radiologist or clinician opinion	0.903	0.903	0.873	0.796
Hu 2022	Arthroscopic or surgical findings			0.847	
Minamoto 2022	Radiologist or clinician opinion	0.860	0.942	0.885	0.910
Shakhovska 2022	Training labels, reports, or annotations		0.855	0.799	
Siouras 2022	Radiologist or clinician opinion				

Azcona 2020	Training labels, reports, or annotations		0.934		
Rizk 2021	Radiologist or clinician opinion	0.860	0.885	0.845	0.780
Sridhar 2022	Training labels, reports, or annotations	0.963		0.954	0.951
Zhang 2020	Arthroscopic or surgical findings	0.944	0.960	0.957	0.976
Fritz 2020	Arthroscopic or surgical findings AND Training labels, reports, or annotations	0.900	0.961	0.850	0.710
Astuto 2021	Radiologist or clinician opinion	0.890	0.930		0.850
Li 2022	Arthroscopic or surgical findings	0.785	0.907	0.924	0.941
Namiri 2020	Radiologist or clinician opinion	0.917		0.920	0.947
Zhu 2022	Radiologist or clinician opinion				
Dung 2023	Training labels, reports, or annotations	0.8966		0.880	0.820
Awan 2021	Training labels, reports, or annotations	0.947	0.980	0.920	0.917
Tran 2022	Training labels, reports, or annotations	0.910	0.942	0.873	0.765
Tack 2021	Training labels, reports, or annotations		0.935		
Pandey 2021	Training labels, reports, or annotations			0.775	
Jeon 2021	Training labels, reports, or annotations	0.975	0.983		0.930
Zhang 2023	Training labels, reports, or annotations			0.997	0.997
Joshi 2022	Arthroscopic or surgical findings AND Training labels, reports, or annotations			0.966	0.967
Li 2023	Radiologist or clinician opinion		0.949		
Berrimi 2024	Non-clinican or non-specialist opinion		0.932	0.904	
Rahouma 2021	Training labels, reports, or annotations			0.880	
Shin 2022	Radiologist or clinician opinion	0.933	0.924	0.920	0.786
Wang 2023	Radiologist or clinician opinion	0.891		0.921	0.960
